# Modulation of LINE-1 and Alu/SVA Retrotransposition by Aicardi-Goutières Syndrome-Related SAMHD1

**DOI:** 10.1016/j.celrep.2013.08.019

**Published:** 2013-09-12

**Authors:** Ke Zhao, Juan Du, Xue Han, John L. Goodier, Peng Li, Xiaohong Zhou, Wei Wei, Sean L. Evans, Linzhang Li, Wenyan Zhang, Ling E. Cheung, Guanjun Wang, Haig H. Kazazian, Xiao-Fang Yu

**Affiliations:** 1 Institute of Virology and AIDS Research, First Hospital of Jilin University, 519 E. Minzhu Avenue, Changchun, Jilin Province 130061, China; 2 School of Life Sciences, Tianjin University, Tianjin 300072, China; 3 Department of Molecular Microbiology and Immunology, Johns Hopkins Bloomberg School of Public Health, 615 N. Wolfe Street, Baltimore, MD 21205, USA; 4 McKusick-Nathans Institute of Genetic Medicine, Johns Hopkins University School of Medicine, Baltimore, MD 21205, USA; 5 Department of Hemotology, First Hospital of Jilin University, 519 E. Minzhu Avenue, Changchun, Jilin Province 130061, China

## Abstract

Long interspersed elements 1 (LINE-1) occupy at least 17% of the human genome and are its only active autonomous retrotransposons. However, the host factors that regulate LINE-1 retrotransposition are not fully understood. Here, we demonstrate that the Aicardi-Goutières syndrome gene product SAMHD1, recently revealed to be an inhibitor of HIV/simian immunodeficiency virus (SIV) infectivity and neutralized by the viral Vpx protein, is also a potent regulator of LINE-1 and LINE-1-mediated Alu/SVA retrotransposition. We also found that mutant SAMHD1s of Aicardi-Goutières syndrome patients are defective in LINE-1 inhibition. Several domains of SAMHD1 are critical for LINE-1 regulation. SAMHD1 inhibits LINE-1 retrotransposition in dividing cells. An enzymatic active site mutant SAMHD1 maintained substantial anti-LINE-1 activity. SAMHD1 inhibits ORF2p-mediated LINE-1 reverse transcription in isolated LINE-1 ribonucleoproteins by reducing ORF2p level. Thus, SAMHD1 may be a cellular regulator of LINE-1 activity that is conserved in mammals.

## INTRODUCTION

LINE-1 is the only active autonomous retroelement in humans and can produce new genomic insertions mediated by its encoded endonuclease (Feng et al., 1996) and reverse transcriptase (Mathias et al., 1991) activities. LINE-1s make up at least 17% of the human genome (Beck et al., 2011; Brouha et al., 2003; Hancks and Kazazian, 2012; Lander et al., 2001). Other nonautonomous retroelements, such as Alu and SVA, complete their retrotransposition process by a mechanism mediated by LINE-1 proteins and account for approximately 1 million and 3,000 copies, respectively (Lander et al., 2001; Ostertag et al., 2003; Wang et al., 2005). Recent data have suggested that the activity of retroelements such as LINE-1, Alu, and SVA can lead to various diseases (Beck et al., 2011; Hancks and Kazazian, 2012).

SAMHD1 mutations can cause Aicardi-Goutières syndrome (AGS), which is characterized as an improper immune activation resulting from the accumulation of intracellular DNA (Rice et al., 2009; Thiele et al., 2010; Xin et al., 2011). SAMHD1 is also a potent cellular restriction factor against retroviruses such as HIV and simian immunodeficiency virus (SIV) (Hrecka et al., 2011; Laguette et al., 2011), with a deoxynucleoside triphosphate triphosphohydrolase (dNTPase) activity linked to retroviral restriction (Goldstone et al., 2011; Kim et al., 2012; Lahouassa et al., 2012; Powell et al., 2011). SAMHD1 inhibits retroviruses in nondividing myeloid cells and resting CD4^+^ T cells by depleting dNTP levels (Baldauf et al., 2012; Descours et al., 2012), although recent studies suggested that the phosphorylation status of SAMHD1 (T592) is also important for the anti-HIV activity (Cribier et al., 2013; White et al., 2013b). However, the ability of SAMHD1 to inhibit endogenous retroelements such as LINE-1 has not been previously reported.

## RESULTS

### The SAMHD1 Protein Is a Potent Cellular Factor Suppressing LINE-1 Activity

**Figure 1:**
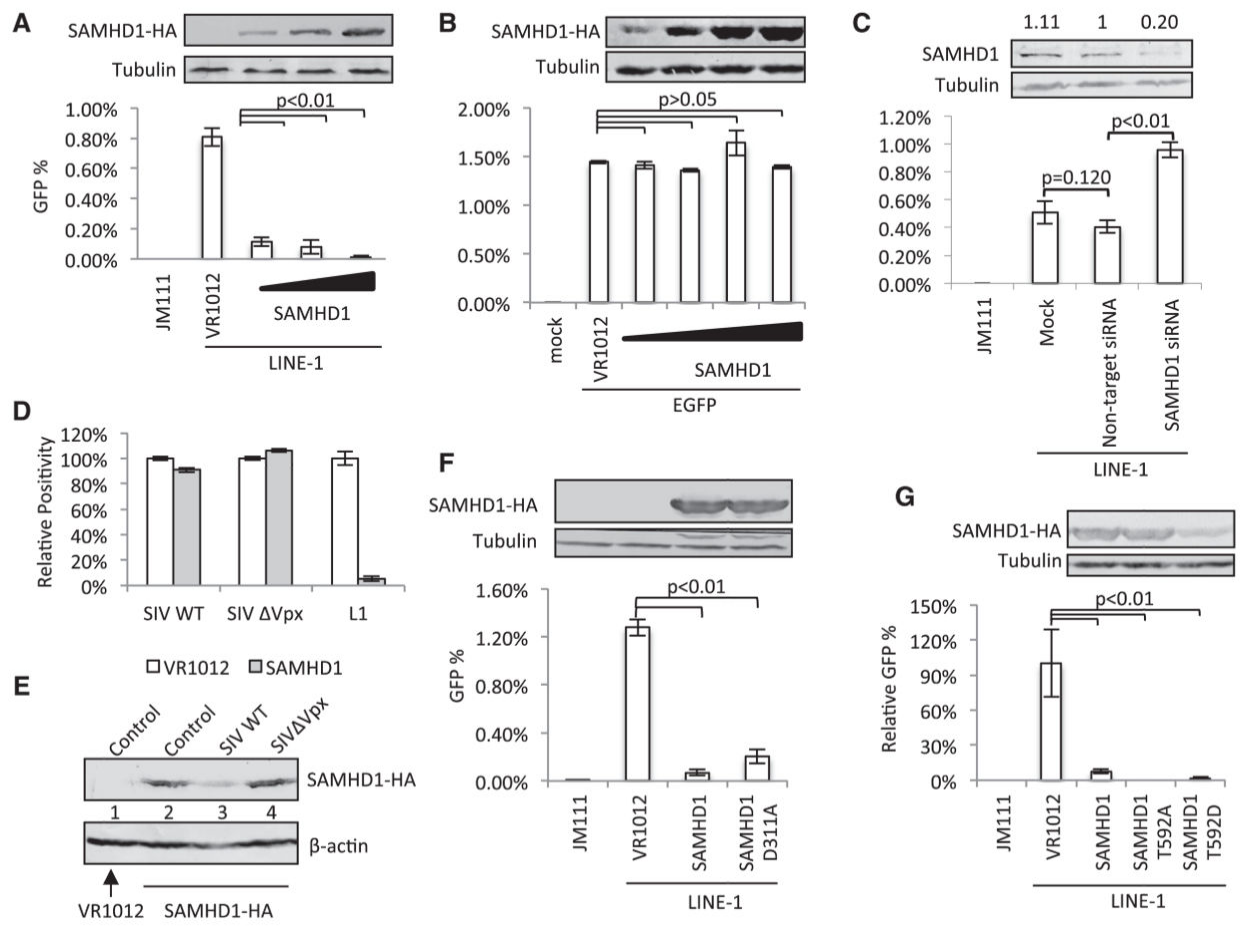
Modulation of LINE-1 Retrotransposition by SAMHD1 (A) SAMHD1 suppressed LINE-1 activity in a dose-dependent manner. 99 PUR RPS EGFP (LINE-1) has been previously described (Moran et al., 1996). The negative control, the defective LINE-1 retrotransposition construct 99 PUR JM111 EGFP (JM111), gave few GFP-positive cells (Figure S1B) and was used as negative control for flow cytometry gating. VR1012 was the empty vector used as negative control for SAMHD1 expression. SAMHD1-expressing plasmids (100, 250, and 500 ng) were cotransfected with LINE-1 into HEK293T cells to examine the possible potency against LINE-1 retrotransposition. EGFP-positive cells were determined by flow cytometry 4 days after transfection. (B) SAMHD1 does not affect CMV promoter-driven expression of EGFP. (C) Depletion of endogenous SAMHD1 with specific siRNA treatment in HEK293T cells enhances LINE-1 activity. The number above the immunoblotting result indicates the relative amount of SAMHD1 mRNA level (the control siRNA-treated sample was set to 1), which was determined by SAMHD1-specific real-time PCR. GAPDH mRNA was also monitored as a cellular mRNA control (data not shown). (D) Transfected SAMHD1 in HEK293T cells inhibits LINE-1 activity but not wild-type SIV or SIVΔVpx infection. HEK293T cells were transfected with the empty vector or SAMHD1 expression vector prior to infection with equal amounts of VSV-G pseudotype SIV WT or SIVΔVpx viruses containing EGFP cassette in viral genome. The transfection efficiency was approximately 90% using similar conditions in a parallel experiment. (E) Transfected SAMHD1-HA in HEK293T cells was depleted during SIV WT but not SIVΔVpx infection. (F) The active site mutant SAMHD1 D311A maintained substantial activity against LINE-1. (G) SAMHD1T592A and SAMHD1T592D maintained LINE-1 inhibition activity. All the data in this figure are representative of at least three independent experiments. The error bars indicated the SD of three replicates within one experiment. See also Figures S1 and S2.

To determine whether LINE-1 is a potential target of SAMHD1, we evaluated the effect of SAMHD1 on LINE-1 retrotransposition using a well-established reporter system in HEK293T cells (Moran et al., 1996; Niewiadomska et al., 2007; Ostertag et al., 2000) (Figure S1A). The LINE-1 construct 99 PUR RPS EGFP contains an EGFP reporter cassette, interrupted by an intron in the opposite transcriptional orientation and inserted into the 3′ UTR of a retrotransposition-competent L1, L1-RP (Kimberland et al., 1999) (Figure S1B). EGFP is expressed only when the LINE-1 transcript is spliced and reverse transcribed, its complementary DNA (cDNA) is inserted into the host genome, and the EGFP reporter gene is expressed from its own CMV promoter. Construct 99 PUR JM111 EGFP (JM111), which contains two missense mutations in ORF1 (Moran et al., 1996), produces no EGFP signal (i.e., it is retrotransposition defective) and was used as a negative control for retrotransposition (Figure S1C). 99 PUR RPS EGFP generated approximately 0.8% GFP-positive cells, whereas JM111 produced no EGFP-positive cells (Figure 1A). The empty vector (VR1012) had no apparent effect on LINE-1 activity when compared to a no-vector control (Figure S1D). SAMHD1 potently inhibited LINE-1 retrotransposition in a dose-dependent fashion in HEK293T cells (Figure 1A). The expression of mCherry had no effect on LINE-1 retrotransposition, whereas fusion protein mCherry-SAMHD1 was still potent in LINE-1 inhibition (Figures S1E and S1F). SAMHD1 expression had no apparent effect on cellular proliferation or cellular β-actin mRNA splicing (Figures S1G and S1H). The LINE-1 assay relies on CMV promoter-driven EGFP expression as a readout. Under a similar percentage of EGFP-positive cells, coexpression of SAMHD1 in a dose manner with a CMV-EGFP-expressing vector did not alter the expression of EGFP (Figure 1B), showing that SAMHD1 does not affect either CMV promoter-mediated transcription or translation/stability of the EGFP protein.

A synthetic human LINE-1 construct (ORFeus-HS) (An et al., 2011) containing codon-modified ORF1 and ORF2 sequences and a deleted 5′ UTR was also suppressed by SAMHD1 (Figures S2A and S2B). Because ORFeus-HS contains little authentic LINE-1 RNA sequence, it appears that SAMHD1 may not target the *cis*-sequence of LINE-1. Furthermore, retrotransposition of Neo-based L1-RP (Kimberland et al., 1999) in HeLa cells was efficiently inhibited by SAMHD1 (Figures S2A, S2C, and S2D). Thus, we have demonstrated that SAMHD1 inhibits retrotrans-position in diverse mammalian LINE-1 systems.

Endogenous SAMHD1 can also function as a LINE-1 inhibitor. Addition of specific small interfering RNAs (siRNAs) targeting SAMHD1 resulted in a 230% increase in LINE-1 retrotransposition in HEK293T cells (Figure 1C). Similar L1 inhibition was observed when the expression of endogenous SAMHD1 was reduced by using a SIV Vpx (viral protein X) expression vector, but not by a mutant VpxQ76A expression vector (defective in mediating SAMHD1 degradation; Wei et al., 2012) in HEK293T cells (Figures S2E and S2F).

Although we achieved approximately 90% transfection efficiency as indicated by the efficient degradation of endogenous SAMHD1 by the transfected Vpx expression vector (Figure S2E) and the detection of mCherry expression after transfection in parallel experiments (data not shown), SAMHD1 expression in HEK293T cells had no obvious effect on the infection of wild-type SIVsmm (SIV-WT) or SIVsmmΔVpx (Figure 1D). It is unlikely that SIV-WT or SIVsmmΔVpx preferentially infected untransfected HEK293T cells, because SIV-WT infection still resulted in transfected SAMHD1 degradation in these cells (Figure 1E). SAMHD1 expression also did not inhibit HIV-1 infection in HEK293T cells (Figure S2G). SAMHD1 restricts the retroviral reverse transcription in nondividing myeloid cells by depleting the intracellular dNTP pool (Berger et al., 2011; Goldstone et al., 2011; Hrecka et al., 2011; Laguette et al., 2011; Lahouassa et al., 2012; Powell et al., 2011). This dNTP depletion activity is countered by the active production of dNTPs in the dividing cells (Manel and Littman, 2011). Our data showing that SAMHD1 cannot suppress HIV/SIV infection in HEK293T cells are consistent with this model. We also observed that SAMHD1 did not inhibit HBV replication (which also requires dNTPs during reverse transcription) in HEK293T cells (Figure S2H). Therefore, SAMHD1 may inhibit LINE-1 and retroviruses through apparently distinct mechanisms. Consistent with this idea, the SAMHD1 mutant D311A, which is defective for dNTPase activity (Gold-stone et al., 2011), maintained the ability to inhibit LINE-1 retro-transposition (Figure 1F). Active site mutants of SAMHD1 have been reported to lack anti-HIV-1 or SIVΔVpx activity (Laguette et al., 2011; Lahouassa et al., 2012). These results suggest that dNTPase activity may not be critical for SAMHD1-mediated LINE-1 inhibition. We have also observed that phosphorylation status of SAMHD1 (T592), which is important for HIV-1 restriction (Cribier et al., 2013; White et al., 2013b), did not affect LINE-1 inhibition (Figure 1G).

### AGS-Related Mutations Compromise the Ability of SAMHD1 to Suppress LINE-1 Activity

**Figure 2:**
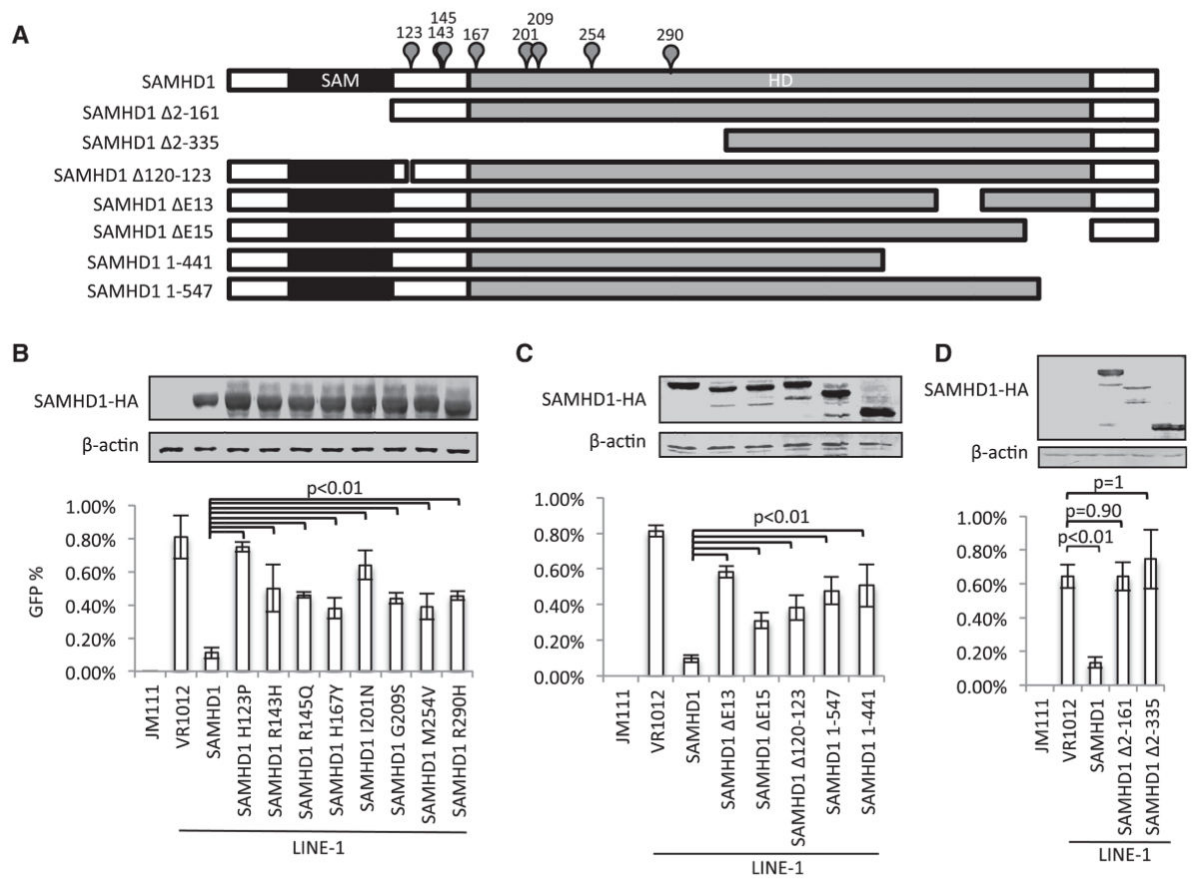
AGS-Related SAMHD1 Mutants Have Reduced Potency against LINE-1 Retrotransposition (A) A diagram indicating SAMHD1 mutant constructs used in this figure. Positions of AGS-related single amino acid mutations are indicated above the wild-type SAMHD1 cartoon. (B) SAMHD1 mutant proteins with single amino acid substitutions detected from AGS patients have compromised activity against LINE-1. (C) Naturally occurring SAMHD1 deletion mutants detected in AGS patients also show reduced LINE-1 inhibition. (D) Both SAMHD1 Δ2-161 and Δ2-335 completely lost their LINE-1 inhibition activity. All the data in this figure are representative of at least three independent experiments. The error bars indicated the SD of three replicates within one experiment. See also Figure S3.

Specific SAMHD1 point mutations, internal deletions, and carboxyl-terminal truncations (Figure 2A) can cause AGS (Dale et al., 2010; Rice et al., 2009; Thiele et al., 2010; Xin et al., 2011). An examination of several SAMHD1 mutants that have been identified in AGS patients indicated that they all show significantly reduced LINE-1 inhibition (p < 0.01 versus wild-type SAMHD1), even when expressed at levels comparable to wild-type SAMHD1 (Figures 2B and 2C). LINE-1 reverse transcription occurs on genomic DNA in the nucleus through a process called target-primed reverse transcription (Luan et al., 1993). SAMHD1 has also been localized to the nucleus (Rice et al., 2009). Thus, nuclear localization could be required for SAMHD1-mediated LINE-1 inhibition. However, we found that the SAMHD1 point mutants and SAMHD1 truncation mutants maintained a nuclear localization in live HEK293T cells (Figure S3A; data not shown).

We further examined regions in SAMHD1 that are important for its anti-LINE-1 activity (Figure 2A). We observed that SAMHD1 mutants lacking the partial HD domain (SAMHD1 Δ162-335) and amino acids 113–136 (SAMHD1 Δ113-136) had weaker activity against LINE-1 than did full-length SAMHD1 (Figures S3B and S3C). On the other hand, deletion of the SAM domain (SAMHD1 Δ42-109 had an increased effect on LINE-1 inhibition (Figures S3B and S3C). However, deletion of the N-terminal region including the linker region abolished SAMHD1-mediated LINE-1 inhibition (Figure 2D). It is worth noting that deletion of SAMHD1 SAM domain reduced its dNTPase and anti-HIV-1 activities (White et al., 2013a). However, SAM domain deletion SAMHD1 mutant still inhibited LINE-1 (Figures S3B and S3C), consistent with the argument that SAMHD1 inhibits LINE-1 and HIV-1/SIV through distinct mechanisms. Thus, in SAMHD1, the linker region between the SAM and HD domains, and possibly the HD domain itself, is critical for LINE1 inhibition. Interestingly, the AGS-related SAMHD1 point mutants that have a reduced capacity for LINE-1 inhibition are all clustered in these regions.

### LINE-1 Suppression Is a Conserved Feature among Mammalian SAMHD1 Proteins

**Figure 3:**
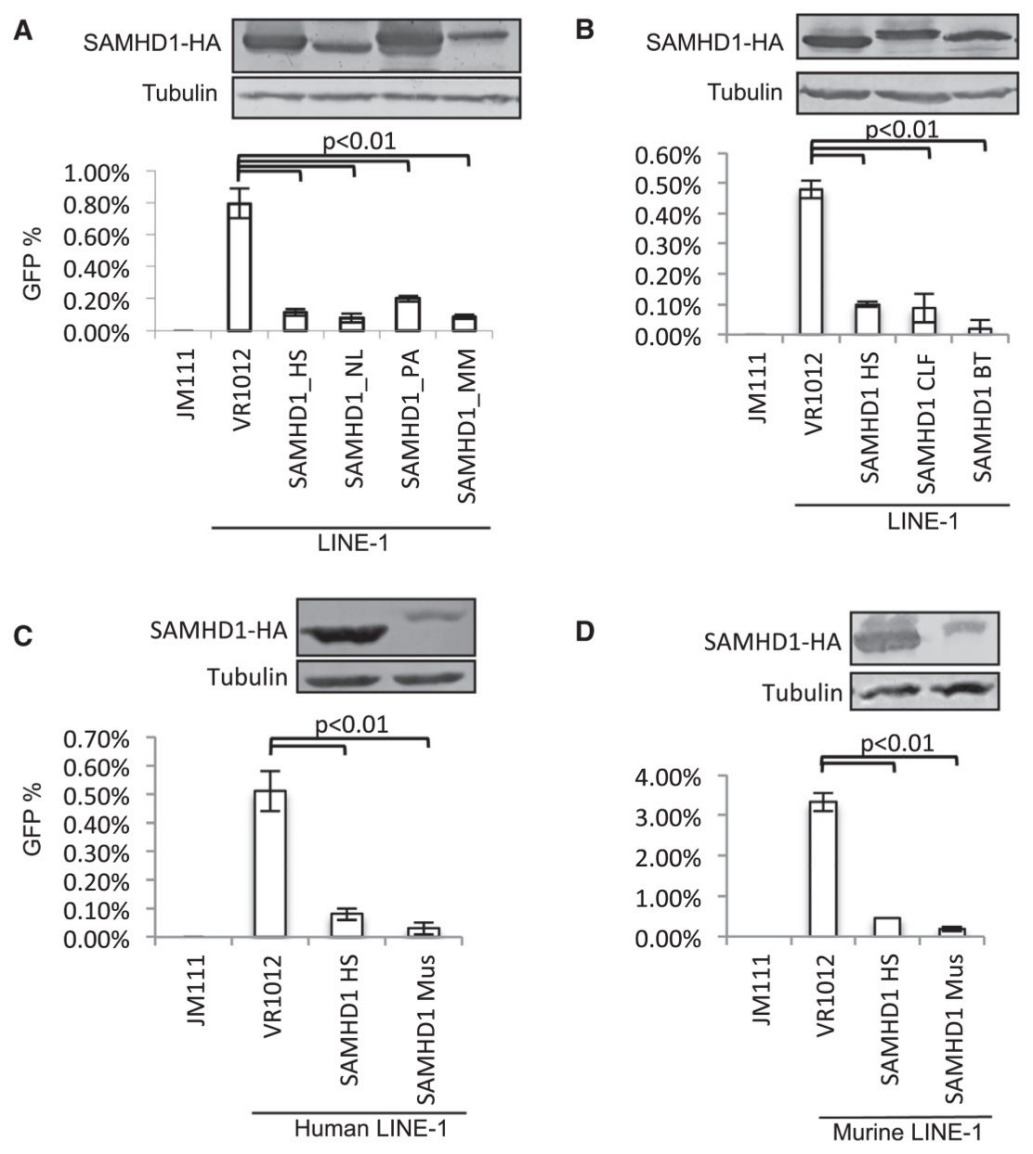
Diverse Mammalian SAMHD1 Proteins Inhibit LINE-1 Retrotransposition (A) Primate SAMHD1 proteins are potent inhibitors of human LINE-1 activity. HS, *Homo sapiens*; NL, *Nomascus leucogenys*; PA, *Pongo abelii*; MM, *Macaca mulatta*. (B) SAMHD1 from mammals shows strong inhibition of human LINE-1, whereas that from chicken does not. CLF, *Canis lupus familiaris*; BT, *Bos Taurus*. (C and D) Comparing to human SAMHD1, greater suppression of either human LINE-1 (Figure 3C) or codon optimized murine LINE-1 (Figure 3D) was observed for murine SAMHD1. Mus, *Mus musculus*. All the data in this figure are representative of at least three independent experiments. The error bars indicated the SD of three replicates within one experiment.

We were also interested in the LINE-1 inhibition potency of SAMHD1 from other animals. SAMHD1 from the nonhuman primates *Nomascus leucogenys* (NL) and *Macaca mulatta* (MM) showed strong LINE-1 inhibition when compared to human SAMHD1 (Figure 3A). SAMHD1 from the primate *Pongo abelii* (PA) had a slightly weaker anti-LINE-1 activity than that of human SAMHD1 (Figure 3A). Also, SAMHD1 from both *Canis lupus familiaris* (CLF) and *Bos taurus* (BT) had strong anti-LINE-1 activity (Figure 3B). Interestingly, human and *Mus musculus* (Mus) SAMHD1 could potently suppress the retrotransposition of LINE-1 from both human and mouse (Figures 3C and 3D). Thus, LINE-1 inhibition appears to be a conserved feature of mammalian SAMHD1 proteins, and SAMHD1 protein functions through a general mechanism to suppress LINE-1 from different species.

### Inhibition of ORF2p-Mediated LINE-1 Reverse Transcription by SAMHD1

**Figure 4:**
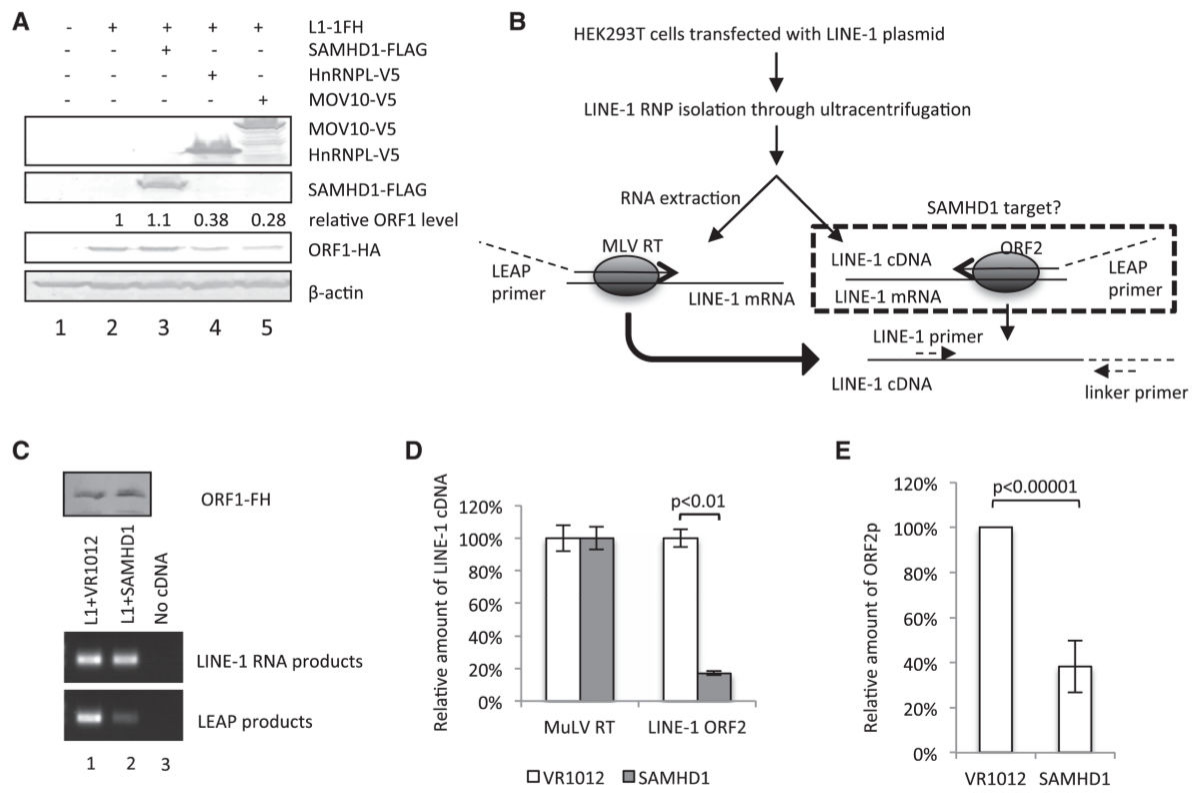
SAMHD1 Inhibits LINE-1 ORF2p-Mediated Endogenous Reverse Transcription in LINE-1 RNP (A) SAMHD1 did not affect the expression of the LINE-1 ORF1 protein. HEK293T cells were transfected with the pc-L1-1FH vector plus the empty vector VR1012 or the expression vector for SAMHD1, HnRNPL, or MOV10. ImageJ software (NIH) was used to quantitate ORF1 band intensities, and their absolute readings are indicated above the immunoblot. (B) A diagram of the LEAP assay. The LINE-1 RNP (from pc-L1-1FH) was produced from transfected HEK293T cells and purified by ultracentrifugation through a sucrose cushion as previously described. The LEAP primer, containing a linker region (dashed line), was used to precisely target onto LINE-1 mRNA, and the reverse transcription occurred with the assistance of the ORF2 protein. Synthesized cDNA was then amplified through standard PCR with two primers (dash arrows) targeting to LINE-1 and the linker. (C) SAMHD1 reduced the reverse transcription efficiency mediated by ORF2p in LINE-1 RNPs. The amount of ORF1 proteins in isolated LINE-1 RNPs in the absence or presence of SAMHD1 was determined by immunoblotting using an anti-HA antibody. LINE-1 RNA was examined by using the LEAP primer and MuLV reverse transcriptase for cDNA synthesis, followed by PCR amplification. (D) Quantitative real-time PCR analysis of LEAP products and LINE-1 RNA RT-PCR products. (E) ORF2p level was lowered by 62% in average, with the presence of exogenous SAMDH1 protein. The bar chart was based on five independent experiments. The error bars indicated the SD. See also Figure S4.

Several attempts were made to identify the potential target of SAMHD1 in LINE-1 retrotransposition. We first tested the effect of SAMHD1 on LINE-1 gene expression. A few transcription factors that interact with LINE-1s have been identified (Becker et al., 1993; Dai et al., 2012; Harris et al., 2009; Tchénio et al., 2000; Yang et al., 2003). HnRNPL (Peddigari et al., 2013) and RNA helicase MOV10 (Goodier et al., 2012) have been reported to influence LINE-1 ORF1 expression. We have confirmed in our assay system that HnRNPL and MOV10 could reduce LINE-1 ORF1p expression (Figure 4A). Unlike HnRNPL and MOV10, SAMHD1 did not alter LINE-1 ORF1p expression (Figure 4A). LINE-1 replication requires reverse transcription of its own RNA genome using ORF2p. An in vitro LEAP reverse transcriptase assay has been developed (Kulpa and Moran, 2006) to assess the function of ORF2p in LINE-1 ribonucleoprotein (RNP) complexes (Figure 4B). LINE-1 RNPs from an ORF1-tagged LINE-1 construct (Goodier et al., 2012) were isolated from transfected HEK293T cells in the absence or presence of SAMHD1. SAMHD1 did not affect the isolation of LINE-1 RNPs, as indicated by the detection of ORF1 protein or LINE-1 RNA (Figure 4C). However, ORF2p-mediated endogenous reverse transcription of LINE-1 RNA (LEAP products) was significantly suppressed in the presence of SAMHD1 (Figure 4C, lane 2) when compared to its absence (lane 1). Quantitative real-time PCR indicated that SAMHD1 caused an 83% reduction of the ORF2-mediated endogenous reverse transcription of LINE-1 RNA (Figure 4D). Further investigation indicated that SAMHD1 reduced the expression of ORF2p, by 62% in average (Figures 4E and S4A). Moreover, SAMHD1 inhibited ORF2p-mediated Alu and SINE-VNTR-Alu (SVA) retrotransposition activity (Figures S4B–S4E).

## DISCUSSION

This study has identified LINE-1, Alu, and SVA retroelements as the potential targets of SAMHD1. We have determined that human SAMHD1 inhibits LINE-1 activity using various established human and mouse LINE-1 retrotransposition assay systems (Moran et al., 1996; Kimberland et al., 1999; Ostertag et al., 2000; Han and Boeke, 2004; An et al., 2011). SAMHD1 can also inhibit the retrotransposition of a modified human synthetic LINE-1 (An et al., 2011) that contains little authentic LINE-1 sequence. Thus, SAMHD1 may not target the LINE-1 *cis*-RNA sequence. Consistent with this argument, we observed no detectable interaction of SAMHD1 with endogenous LINE-1 RNA, although LINE-1 RNA binding with ORF1 protein was readily detected (data not shown).

The effect of SAMHD1 on LINE-1 activity appeared to be different from other cellular regulators such as HnRNPL and MOV10. HnRNPL (Peddigari et al., 2013) and MOV10 (Goodier et al., 2012) have been observed to affect LINE-1 ORF1 expression. On the other hand, we have observed that SAMHD1 did not alter LINE-1 ORF1p expression (Figure 4A). However, SAMHD1 reduced ORF2p expression and suppressed ORF2p-mediated LINE-1 reverse transcription in purified LINE-1 RNP. Furthermore, SAMHD1 also inhibited ORF2p-mediated Alu and SVA retrotransposition. Thus, LINE-1 ORF2p may be a potential target of SAMHD1.

Interestingly, it seems that SAMHD1 may inhibit retroviruses and retrotransposons through different mechanisms. SAMHD1 inhibits HIV/SIV in nondividing but not dividing cells. In contrast, LINE-1 inhibition by SAMHD1 is observed in dividing cells. SAMHD1’s dNTPase activity depends on residues H167, H206, D207, and D311 within the HD domain (Aravind and Koonin, 1998; Goldstone et al., 2011; Powell et al., 2011) and is crucial for retroviral inhibition because mutations at H206/D207 compromise both enzymatic activity and antiviral potency (Kim et al., 2012; Laguette et al., 2011; Lahouassa et al., 2012). However, in our study, the D311A mutation, which depletes the hydrolase activity of SAMHD1 (Goldstone et al., 2011), still functions as a LINE-1 inhibitor. Elucidating such a mechanism would provide additional critical information to increase our understanding of the process of retrotransposition, as well as providing a potential explanation for SAMHD1 mutations leading to AGS.

Thus far, five genes (*TREX1*, *RNASEH2A*, *RNASEH2B*, *RNASEH2C*, and *SAMHD1*) have been linked to AGS and are related to an inability to remove cellular nucleotide debris intracellularly (Ali et al., 2006; Crow et al., 2006; Rice et al., 2009; Thiele et al., 2010; Xin et al., 2011). There is no direct link between LINE-1 activity and the cellular immune response; however, it has recently been suggested that LINE-1 activity is linked to both interferon expression and selected autoimmune disorders (Crow, 2010; Mavragani and Crow, 2010). A recent report concluded that LINE-1 activity is upregulated in *TREX1* knockout cells, suggesting that TREX1 is a LINE-1 inhibitor (Stetson et al., 2008). Here, we provide evidence that another AGS-related protein, SAMHD1, is also a potent LINE-1 suppressor. AGS-related mutations compromise the potency of SAMHD1 against LINE-1 retrotransposition. AGS-related mutations also compromised TREX1’s potency against LINE-1 (Stetson et al., 2008). Interestingly, reverse transcriptase inhibitors could reduce disease symptoms generated in *TREX1*-knockout mice (Beck-Engeser et al., 2011). Further investigation is required to understand the relationship between retroelements such as LINE-1, AGS-suspected genes, and AGS itself.

## EXPERIMENTAL PROCEDURES

### Cell Culture and LINE-1 Retrotransposition Assay

HEK293T cells and HeLa-HA cells were grown in DMEM medium with 10% FBS (HyClone), GlutaMax, and Penstrep (Invitrogen). All transfections used Lipofectamine 2000 (Invitrogen) reagent. HeLa-HA cells were a gift from J.V. Moran (University of Michigan).

The human L1 plasmids 99 PUR L1RP EGFP (L1) and 99 PUR JM111 EGFP (JM111, as a negative control) have been previously described (Ostertag et al., 2000). Similar protocols were also applied to either the synthetic human LINE-1 sL1-ORFeus-HS (An et al., 2011) or the murine LINE-1 plasmid ORFeus (gifts of Dr. J.D. Boeke) (Han and Boeke, 2004). In brief, LINE-1 plasmid was transfected into HEK293T cells at 2 μg per well in 12-well plates, together with VR1012 or one of the test plasmids. The cells were selected by the addition of puromycin (final concentration, 5 μg/ml) at 48 hr posttransfection. GFP-positive cells were examined 48 hr later by flow cytometry using FACSCalibur. Gating exclusions were based on background fluorescence of the plasmid 99 PUR JM111 EGFP, an L1 construct containing two point mutations in ORF1 that completely abolish retrotransposition; 10,000 single-cell events per sample were gated and analyzed using CellQuest Pro (v.5.2). For the mneoI-based LINE-1 retrotransposition assay, 1 μg LcRPS-mneoI was cotransfected in 6-well plates with 0.5 μg empty vector or the SAMHD1 expression vector into HEK293T cells. At 4 days posttransfection, selection with G418 was initiated and continued for 13 days. The cells in T75 flasks were fixed with PBS/paraformaldehyde/glutaraldehyde, and colonies stained with 0.4% Giemsa.

### LEAP Assays and RT–PCR

The LINE-1 construct containing FLAG-HA-tagged ORF1, pc-L1-1FH, has been described (Goodier et al., 2012). It was cotransfected in the absence or presence of the SAMHD1 expression vector into HEK293T cells. At 2 days after transfection, LINE-1 RNPs were isolated by ultracentrifugation through a sucrose cushion as previously described (Kulpa and Moran, 2006). The LINE-1 RNP sample (2 μl) was added to each cDNA extension reaction (LEAP) as previously described (Kulpa and Moran, 2006), using the 3′ RACE adaptor NV: 5′-GCGAGCACAGAATTAATACGACTCACTATAGG TTTTTTTTTTTTVN-3′ as primer. Also, LINE-1 RNA was extracted from the LINE-1 RNP, treated with TURBO DNase (Invitrogen), and reverse transcribed using 3′ RACE adaptor NV as primer and MuLV RT with a High Capacity cDNA Reverse Transcription Kit (ABI Applied Biosystems). PCR was performed as previously described (Kulpa and Moran, 2006). PCR products were separated on 2% agarose gels and visualized by the Red Personal Gel Imaging System (ProteinSimple). PCR products were also sequenced and found to match the expected LINE-1 sequence. The relative amount of synthesized cDNA from both methods were then determined by real-time PCR using the primers Linker (as part of primer 3′ RACE adaptor NV), 5′-GCGAGCACAGAATTAATACGACT-3′; L1-LEAP-R, 5′-GGGTTCGAAATCGATAAGCTTGGATCCAGAC-3′, with a standard two step method (95°C for 15 s and 60°C for 1 min) with a cycle number of 40. See also Extended Experimental Procedures.

## Supplementary Material

1
