# Supplementary material for: Modulation of LINE-1 and Alu/SVA Retrotransposition by Aicardi-Goutières Syndrome-Related SAMHD1
Source: Cell Rep. Author manuscript; Available in PMC 2014 Sep 26. (PMC3988314; doi:10.1016/j.celrep.2013.08.019)
Supplement: 1 [file NIHMS520731-supplement-1.pdf]

Cell PRESS
Open ACCESS
Cell Reports
Report

Modulation of LINE-1 and Alu/SVA Retrotransposition by Aicardi-Goutières Syndrome-Related SAMHD1

Ke Zhao,[1] Juan Du,[1] Xue Han,[2,3] John L. Goodier,[4] Peng Li,[1] Xiaohong Zhou,[2,3] Wei Wei,[1] Sean L. Evans,[3] Linzhang Li,[1] Wenyan Zhang,[1] Ling E. Cheung,[4] Guanjun Wang,[5] Haig H. Kazazian, Jr.,[4] and Xiao-Fang Yu[1,2,3,*]
[1]Institute of Virology and AIDS Research, First Hospital of Jilin University, 519 E. Minzhu Avenue, Changchun, Jilin Province 130061, China
[2]School of Life Sciences, Tianjin University, Tianjin 300072, China
[3]Department of Molecular Microbiology and Immunology, Johns Hopkins Bloomberg School of Public Health, 615 N. Wolfe Street, Baltimore, MD 21205, USA
[4]McKusick-Nathans Institute of Genetic Medicine, Johns Hopkins University School of Medicine, Baltimore, MD 21205, USA
[5]Department of Hemotology, First Hospital of Jilin University, 519 E. Minzhu Avenue, Changchun, Jilin Province 130061, China
*Correspondence: xfyu@jhsph.edu
http://dx.doi.org/10.1016/j.celrep.2013.08.019
This is an open-access article distributed under the terms of the Creative Commons Attribution License, which permits unrestricted use, distribution, and reproduction in any medium, provided the original author and source are credited.

SUMMARY

Long interspersed elements 1 (LINE-1) occupy at least 17% of the human genome and are its only active autonomous retrotransposons. However, the host factors that regulate LINE-1 retrotransposition are not fully understood. Here, we demonstrate that the Aicardi-Goutières syndrome gene product SAMHD1, recently revealed to be an inhibitor of HIV/simian immunodeficiency virus (SIV) infectivity and neutralized by the viral Vpx protein, is also a potent regulator of LINE-1 and LINE-1-mediated Alu/SVA retrotransposition. We also found that mutant SAMHD1s of Aicardi-Goutières syndrome patients are defective in LINE-1 inhibition. Several domains of SAMHD1 are critical for LINE-1 regulation. SAMHD1 inhibits LINE-1 retrotransposition in dividing cells. An enzymatic active site mutant SAMHD1 maintained substantial anti-LINE-1 activity. SAMHD1 inhibits ORF2p-mediated LINE-1 reverse transcription in isolated LINE-1 ribonucleoproteins by reducing ORF2p level. Thus, SAMHD1 may be a cellular regulator of LINE-1 activity that is conserved in mammals.

INTRODUCTION

LINE-1 is the only active autonomous retroelement in humans and can produce new genomic insertions mediated by its encoded endonuclease (Feng et al., 1996) and reverse transcriptase (Mathias et al., 1991) activities. LINE-1s make up at least 17% of the human genome (Beck et al., 2011; Brouha et al., 2003; Hancks and Kazazian, 2012; Lander et al., 2001). Other nonautonomous retroelements, such as Alu and SVA, complete their retrotransposition process by a mechanism mediated by LINE-1 proteins and account for approximately 1 million and 3,000 copies, respectively (Lander et al., 2001; Ostertag et al., 2003; Wang et al., 2005). Recent data have suggested that the activity of retroelements such as LINE-1, Alu, and SVA can lead to various diseases (Beck et al., 2011; Hancks and Kazazian, 2012).

SAMHD1 mutations can cause Aicardi-Goutières syndrome (AGS), which is characterized as an improper immune activation resulting from the accumulation of intracellular DNA (Rice et al., 2009; Thiele et al., 2010; Xin et al., 2011). SAMHD1 is also a potent cellular restriction factor against retroviruses such as HIV and simian immunodeficiency virus (SIV) (Hrecka et al., 2011; Laguette et al., 2011), with a deoxynucleoside triphosphate triphosphohydrolase (dNTPase) activity linked to retroviral restriction (Goldstone et al., 2011; Kim et al., 2012; Lahouassa et al., 2012; Powell et al., 2011). SAMHD1 inhibits retroviruses in nondividing myeloid cells and resting $CD4^+$ T cells by depleting dNTP levels (Baldauf et al., 2012; Descours et al., 2012), although recent studies suggested that the phosphorylation status of SAMHD1 (T592) is also important for the anti-HIV activity (Cribier et al., 2013; White et al., 2013b). However, the ability of SAMHD1 to inhibit endogenous retroelements such as LINE-1 has not been previously reported.

RESULTS

The SAMHD1 Protein Is a Potent Cellular Factor Suppressing LINE-1 Activity

To determine whether LINE-1 is a potential target of SAMHD1, we evaluated the effect of SAMHD1 on LINE-1 retrotransposition using a well-established reporter system in HEK293T cells (Moran et al., 1996; Niewiadomska et al., 2007; Ostertag et al., 2000) (Figure S1A). The LINE-1 construct 99 PUR RPS EGFP contains an EGFP reporter cassette, interrupted by an intron in the opposite transcriptional orientation and inserted into the 3′ UTR of a retrotransposition-competent L1, L1-RP (Kimberland et al., 1999) (Figure S1B). EGFP is expressed only when the

CrossMark

Cell PRESS Open ACCESS

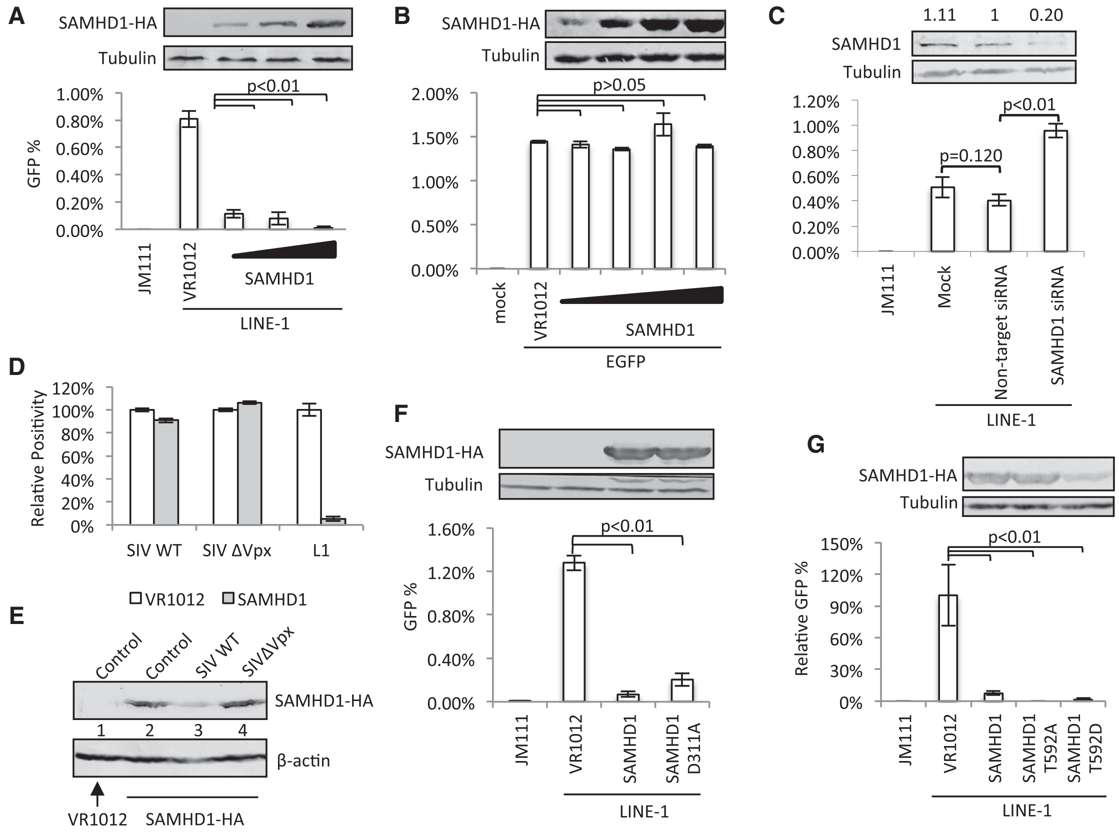

Figure 1. Modulation of LINE-1 Retrotransposition by SAMHD1

(A) SAMHD1 suppressed LINE-1 activity in a dose-dependent manner. 99 PUR RPS EGFP (LINE-1) has been previously described (Moran et al., 1996). The negative control, the defective LINE-1 retrotransposition construct 99 PUR JM111 EGFP (JM111), gave few GFP-positive cells (Figure S1B) and was used as negative control for flow cytometry gating. VR1012 was the empty vector used as negative control for SAMHD1 expression. SAMHD1-expressing plasmids (100, 250, and 500 ng) were cotransfected with LINE-1 into HEK293T cells to examine the possible potency against LINE-1 retrotransposition. EGFP-positive cells were determined by flow cytometry 4 days after transfection.

(B) SAMHD1 does not affect CMV promoter-driven expression of EGFP.

(C) Depletion of endogenous SAMHD1 with specific siRNA treatment in HEK293T cells enhances LINE-1 activity. The number above the immunoblotting result indicates the relative amount of SAMHD1 mRNA level (the control siRNA-treated sample was set to 1), which was determined by SAMHD1-specific real-time PCR. GAPDH mRNA was also monitored as a cellular mRNA control (data not shown).

(D) Transfected SAMHD1 in HEK293T cells inhibits LINE-1 activity but not wild-type SIV or SIVΔVpx infection. HEK293T cells were transfected with the empty vector or SAMHD1 expression vector prior to infection with equal amounts of VSV-G pseudotype SIV WT or SIVΔVpx viruses containing EGFP cassette in viral genome. The transfection efficiency was approximately 90% using similar conditions in a parallel experiment.

(E) Transfected SAMHD1-HA in HEK293T cells was depleted during SIV WT but not SIVΔVpx infection.

(F) The active site mutant SAMHD1 D311A maintained substantial activity against LINE-1.

(G) SAMHD1T592A and SAMHD1T592D maintained LINE-1 inhibition activity. All the data in this figure are representative of at least three independent experiments. The error bars indicated the SD of three replicates within one experiment.

See also Figures S1 and S2.

LINE-1 transcript is spliced and reverse transcribed, its complementary DNA (cDNA) is inserted into the host genome, and the EGFP reporter gene is expressed from its own CMV promoter. Construct 99 PUR JM111 EGFP (JM111), which contains two missense mutations in ORF1 (Moran et al., 1996), produces no EGFP signal (i.e., it is retrotransposition defective) and was used as a negative control for retrotransposition (Figure S1C). 99 PUR RPS EGFP generated approximately 0.8% GFP-positive cells, whereas JM111 produced no EGFP-positive cells (Figure 1A). The empty vector (VR1012) had no apparent effect on LINE-1 activity when compared to a no-vector control (Figure S1D). SAMHD1 potently inhibited LINE-1 retrotransposition in a dose-dependent fashion in HEK293T cells (Figure 1A). The expression of mCherry had no effect on LINE-1 retrotransposition, whereas fusion protein mCherry-SAMHD1 was still potent in LINE-1 inhibition (Figures S1E and S1F). SAMHD1 expression had no apparent effect on cellular proliferation or cellular β-actin mRNA splicing (Figures S1G and S1H). The LINE-1 assay relies on CMV promoter-driven EGFP expression as a readout. Under a similar percentage of EGFP-positive cells, coexpression of SAMHD1 in a dose manner with a CMV-EGFP-expressing vector did not alter the expression of EGFP (Figure 1B), showing that SAMHD1 does not affect either CMV promoter-mediated transcription or translation/stability of the EGFP protein.

A synthetic human LINE-1 construct (ORFeus-HS) (An et al., 2011) containing codon-modified ORF1 and ORF2 sequences and a deleted 5′ UTR was also suppressed by SAMHD1 (Figures S2A and S2B). Because ORFeus-HS contains little authentic LINE-1 RNA sequence, it appears that SAMHD1 may not target the *cis*-sequence of LINE-1. Furthermore, retrotransposition of Neo-based L1-RP (Kimberland et al., 1999) in HeLa cells was efficiently inhibited by SAMHD1 (Figures S2A, S2C, and S2D). Thus, we have demonstrated that SAMHD1 inhibits retrotransposition in diverse mammalian LINE-1 systems.

Endogenous SAMHD1 can also function as a LINE-1 inhibitor. Addition of specific small interfering RNAs (siRNAs) targeting SAMHD1 resulted in a 230% increase in LINE-1 retrotransposition in HEK293T cells (Figure 1C). Similar L1 inhibition was observed when the expression of endogenous SAMHD1 was reduced by using a SIV Vpx (viral protein X) expression vector, but not by a mutant VpxQ76A expression vector (defective in mediating SAMHD1 degradation; Wei et al., 2012) in HEK293T cells (Figures S2E and S2F).

Although we achieved approximately 90% transfection efficiency as indicated by the efficient degradation of endogenous SAMHD1 by the transfected Vpx expression vector (Figure S2E) and the detection of mCherry expression after transfection in parallel experiments (data not shown), SAMHD1 expression in HEK293T cells had no obvious effect on the infection of wild-type SIVsmm (SIV-WT) or SIVsmmΔVpx (Figure 1D). It is unlikely that SIV-WT or SIVsmmΔVpx preferentially infected untransfected HEK293T cells, because SIV-WT infection still resulted in transfected SAMHD1 degradation in these cells (Figure 1E). SAMHD1 expression also did not inhibit HIV-1 infection in HEK293T cells (Figure S2G). SAMHD1 restricts the retroviral reverse transcription in nondividing myeloid cells by depleting the intracellular dNTP pool (Berger et al., 2011; Goldstone et al., 2011; Hrecka et al., 2011; Laguette et al., 2011; Lahouassa et al., 2012; Powell et al., 2011). This dNTP depletion activity is countered by the active production of dNTPs in the dividing cells (Manel and Littman, 2011). Our data showing that SAMHD1 cannot suppress HIV/SIV infection in HEK293T cells are consistent with this model. We also observed that SAMHD1 did not inhibit HBV replication (which also requires dNTPs during reverse transcription) in HEK293T cells (Figure S2H). Therefore, SAMHD1 may inhibit LINE-1 and retroviruses through apparently distinct mechanisms. Consistent with this idea, the SAMHD1 mutant D311A, which is defective for dNTPase activity (Goldstone et al., 2011), maintained the ability to inhibit LINE-1 retrotransposition (Figure 1F). Active site mutants of SAMHD1 have been reported to lack anti-HIV-1 or SIVΔVpx activity (Laguette et al., 2011; Lahouassa et al., 2012). These results suggest that dNTPase activity may not be critical for SAMHD1-mediated LINE-1 inhibition. We have also observed that phosphorylation status of SAMHD1 (T592), which is important for HIV-1 restriction (Cribier et al., 2013; White et al., 2013b), did not affect LINE-1 inhibition (Figure 1G).

AGS-Related Mutations Compromise the Ability of SAMHD1 to Suppress LINE-1 Activity

Specific SAMHD1 point mutations, internal deletions, and carboxyl-terminal truncations (Figure 2A) can cause AGS (Dale et al., 2010; Rice et al., 2009; Thiele et al., 2010; Xin et al., 2011). An examination of several SAMHD1 mutants that have been identified in AGS patients indicated that they all show significantly reduced LINE-1 inhibition ($p < 0.01$ versus wild-type SAMHD1), even when expressed at levels comparable to wild-type SAMHD1 (Figures 2B and 2C). LINE-1 reverse transcription occurs on genomic DNA in the nucleus through a process called target-primed reverse transcription (Luan et al., 1993). SAMHD1 has also been localized to the nucleus (Rice et al., 2009). Thus, nuclear localization could be required for SAMHD1-mediated LINE-1 inhibition. However, we found that the SAMHD1 point mutants and SAMHD1 truncation mutants maintained a nuclear localization in live HEK293T cells (Figure S3A; data not shown).

We further examined regions in SAMHD1 that are important for its anti-LINE-1 activity (Figure 2A). We observed that SAMHD1 mutants lacking the partial HD domain (SAMHD1 Δ162-335) and amino acids 113–136 (SAMHD1 Δ113-136) had weaker activity against LINE-1 than did full-length SAMHD1 (Figures S3B and S3C). On the other hand, deletion of the SAM domain (SAMHD1 Δ42-109 had an increased effect on LINE-1 inhibition (Figures S3B and S3C). However, deletion of the N-terminal region including the linker region abolished SAMHD1-mediated LINE-1 inhibition (Figure 2D). It is worth noting that deletion of SAMHD1 SAM domain reduced its dNTPase and anti-HIV-1 activities (White et al., 2013a). However, SAM domain deletion SAMHD1 mutant still inhibited LINE-1 (Figures S3B and S3C), consistent with the argument that SAMHD1 inhibits LINE-1 and HIV-1/SIV through distinct mechanisms. Thus, in SAMHD1, the linker region between the SAM and HD domains, and possibly the HD domain itself, is critical for LINE1 inhibition. Interestingly, the AGS-related SAMHD1 point mutants that have a reduced capacity for LINE-1 inhibition are all clustered in these regions.

LINE-1 Suppression Is a Conserved Feature among Mammalian SAMHD1 Proteins

We were also interested in the LINE-1 inhibition potency of SAMHD1 from other animals. SAMHD1 from the nonhuman primates *Nomascus leucogenys* (NL) and *Macaca mulatta* (MM) showed strong LINE-1 inhibition when compared to human SAMHD1 (Figure 3A). SAMHD1 from the primate *Pongo abelii* (PA) had a slightly weaker anti-LINE-1 activity than that of human SAMHD1 (Figure 3A). Also, SAMHD1 from both *Canis lupus familiaris* (CLF) and *Bos taurus* (BT) had strong anti-LINE-1 activity (Figure 3B). Interestingly, human and *Mus musculus* (Mus) SAMHD1 could potently suppress the retrotransposition of LINE-1 from both human and mouse (Figures 3C and 3D). Thus, LINE-1 inhibition appears to be a conserved feature of mammalian SAMHD1 proteins, and SAMHD1 protein functions through a general mechanism to suppress LINE-1 from different species.

Inhibition of ORF2p-Mediated LINE-1 Reverse Transcription by SAMHD1

Several attempts were made to identify the potential target of SAMHD1 in LINE-1 retrotransposition. We first tested the effect of SAMHD1 on LINE-1 gene expression. A few transcription factors that interact with LINE-1s have been identified (Becker et al.,

Cell PRESS
Open ACCESS

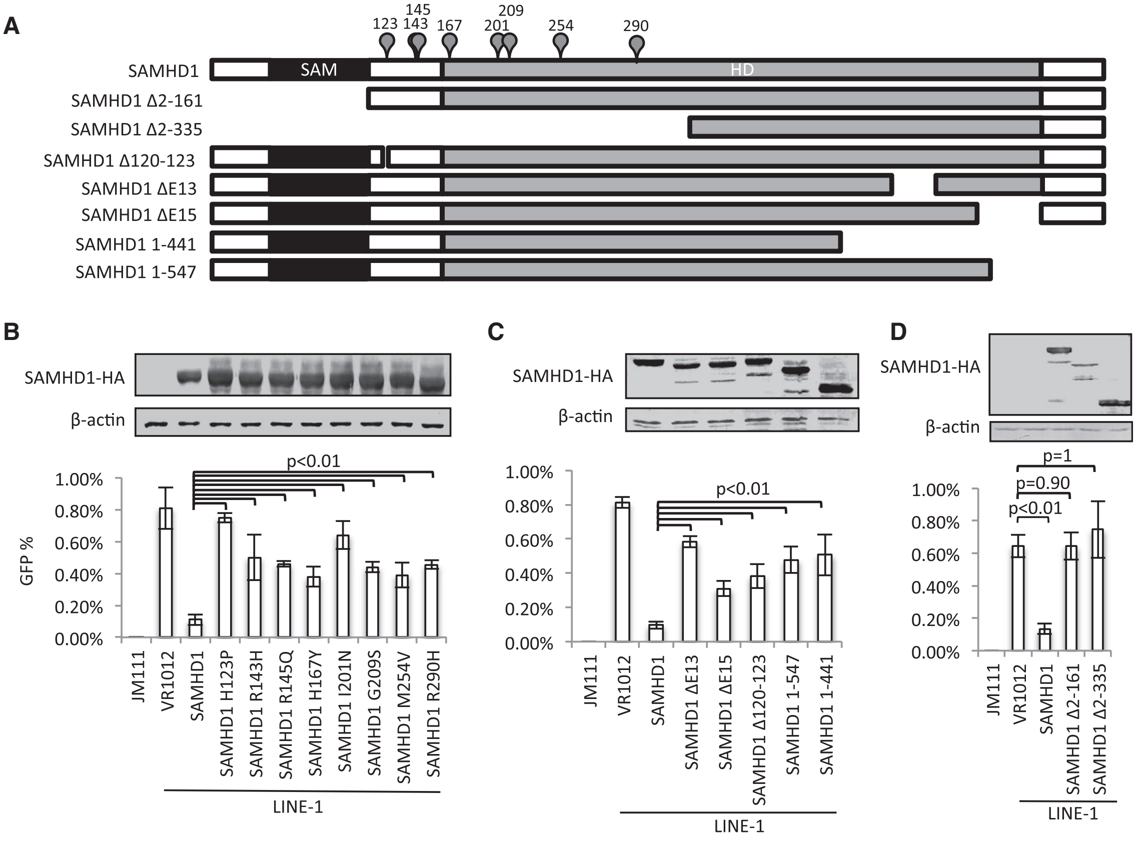

Figure 2. AGS-Related SAMHD1 Mutants Have Reduced Potency against LINE-1 Retrotransposition

(A) A diagram indicating SAMHD1 mutant constructs used in this figure. Positions of AGS-related single amino acid mutations are indicated above the wild-type SAMHD1 cartoon.

(B) SAMHD1 mutant proteins with single amino acid substitutions detected from AGS patients have compromised activity against LINE-1.

(C) Naturally occurring SAMHD1 deletion mutants detected in AGS patients also show reduced LINE-1 inhibition.

(D) Both SAMHD1 Δ2-161 and Δ2-335 completely lost their LINE-1 inhibition activity. All the data in this figure are representative of at least three independent experiments. The error bars indicated the SD of three replicates within one experiment.

See also Figure S3.

1993; Dai et al., 2012; Harris et al., 2009; Tchénio et al., 2000; Yang et al., 2003). HnRNPL (Peddigari et al., 2013) and RNA helicase MOV10 (Goodier et al., 2012) have been reported to influence LINE-1 ORF1 expression. We have confirmed in our assay system that HnRNPL and MOV10 could reduce LINE-1 ORF1p expression (Figure 4A). Unlike HnRNPL and MOV10, SAMHD1 did not alter LINE-1 ORF1p expression (Figure 4A). LINE-1 replication requires reverse transcription of its own RNA genome using ORF2p. An in vitro LEAP reverse transcriptase assay has been developed (Kulpa and Moran, 2006) to assess the function of ORF2p in LINE-1 ribonucleoprotein (RNP) complexes (Figure 4B). LINE-1 RNPs from an ORF1-tagged LINE-1 construct (Goodier et al., 2012) were isolated from transfected HEK293T cells in the absence or presence of SAMHD1. SAMHD1 did not affect the isolation of LINE-1 RNPs, as indicated by the detection of ORF1 protein or LINE-1 RNA (Figure 4C). However, ORF2p-mediated endogenous reverse transcription of LINE-1 RNA (LEAP products) was significantly suppressed in the presence of SAMHD1 (Figure 4C, lane 2) when compared to its absence (lane 1). Quantitative real-time PCR indicated that SAMHD1 caused an 83% reduction of the ORF2-mediated endogenous reverse transcription of LINE-1 RNA (Figure 4D). Further investigation indicated that SAMHD1 reduced the expression of ORF2p, by 62% in average (Figures 4E and S4A). Moreover, SAMHD1 inhibited ORF2p-mediated Alu and SINE-VNTR-Alu (SVA) retrotransposition activity (Figures S4B–S4E).

DISCUSSION

This study has identified LINE-1, Alu, and SVA retroelements as the potential targets of SAMHD1. We have determined that human SAMHD1 inhibits LINE-1 activity using various established human and mouse LINE-1 retrotransposition assay systems (Moran et al., 1996; Kimberland et al., 1999; Ostertag et al., 2000; Han and Boeke, 2004; An et al., 2011). SAMHD1 can also inhibit the retrotransposition of a modified human synthetic LINE-1 (An et al., 2011) that contains little authentic LINE-1 sequence. Thus, SAMHD1 may not target the LINE-1 *cis*-RNA sequence. Consistent with this argument, we observed no detectable interaction of SAMHD1 with endogenous LINE-1

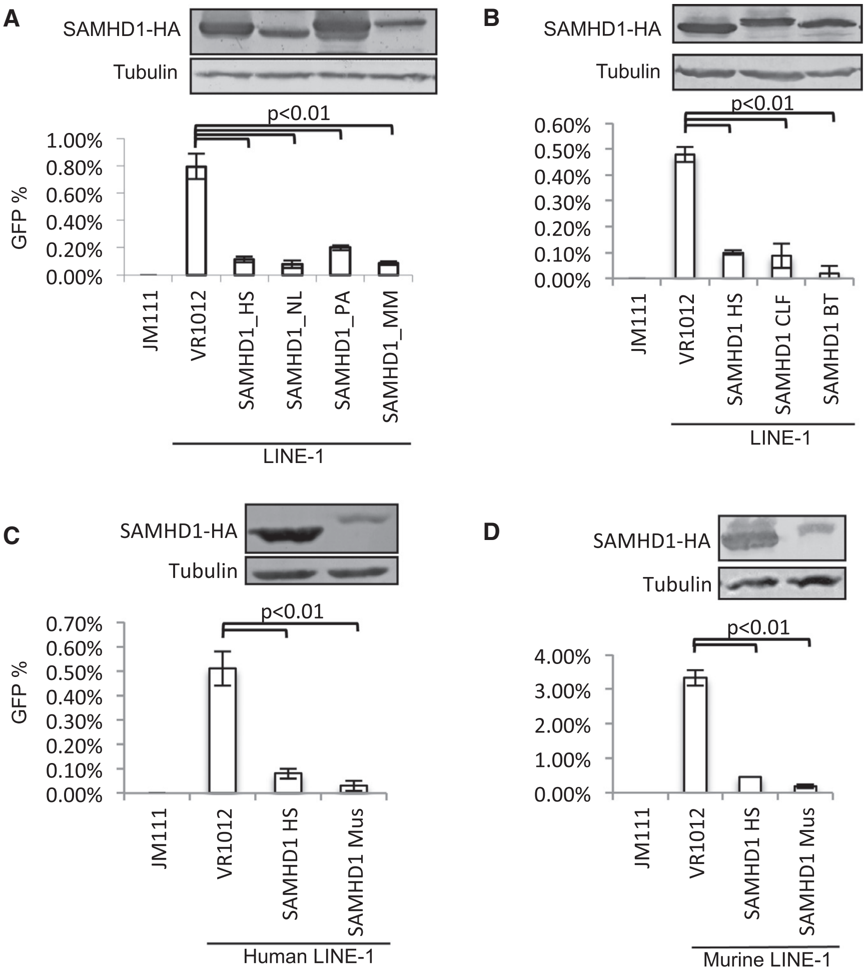

Figure 3. Diverse Mammalian SAMHD1 Proteins Inhibit LINE-1 Retrotransposition

(A) Primate SAMHD1 proteins are potent inhibitors of human LINE-1 activity. HS, *Homo sapiens*; NL, *Nomascus leucogenys*; PA, *Pongo abelii*; MM, *Macaca mulatta*.

(B) SAMHD1 from mammals shows strong inhibition of human LINE-1, whereas that from chicken does not. CLF, *Canis lupus familiaris*; BT, *Bos Taurus*.

(C and D) Comparing to human SAMHD1, greater suppression of either human LINE-1 (Figure 3C) or codon optimized murine LINE-1 (Figure 3D) was observed for murine SAMHD1. Mus, *Mus musculus*. All the data in this figure are representative of at least three independent experiments. The error bars indicated the SD of three replicates within one experiment.

RNA, although LINE-1 RNA binding with ORF1 protein was readily detected (data not shown).

The effect of SAMHD1 on LINE-1 activity appeared to be different from other cellular regulators such as HnRNPL and MOV10. HnRNPL (Peddigari et al., 2013) and MOV10 (Goodier et al., 2012) have been observed to affect LINE-1 ORF1 expression. On the other hand, we have observed that SAMHD1 did not alter LINE-1 ORF1p expression (Figure 4A). However, SAMHD1 reduced ORF2p expression and suppressed ORF2p-mediated LINE-1 reverse transcription in purified LINE-1 RNP. Furthermore, SAMHD1 also inhibited ORF2p-mediated Alu and SVA retrotransposition. Thus, LINE-1 ORF2p may be a potential target of SAMHD1.

Interestingly, it seems that SAMHD1 may inhibit retroviruses and retrotransposons through different mechanisms. SAMHD1 inhibits HIV/SIV in nondividing but not dividing cells. In contrast, LINE-1 inhibition by SAMHD1 is observed in dividing cells. SAMHD1's dNTPase activity depends on residues H167, H206, D207, and D311 within the HD domain (Aravind and Koonin, 1998; Goldstone et al., 2011; Powell et al., 2011) and is crucial for retroviral inhibition because mutations at H206/D207 compromise both enzymatic activity and antiviral potency (Kim et al., 2012; Laguette et al., 2011; Lahouassa et al., 2012). However, in our study, the D311A mutation, which depletes the hydrolase activity of SAMHD1 (Goldstone et al., 2011), still functions as a LINE-1 inhibitor. Elucidating such a mechanism would provide additional critical information to increase our understanding of the process of retrotransposition, as well as providing a potential explanation for SAMHD1 mutations leading to AGS.

Thus far, five genes (*TREX1*, *RNASEH2A*, *RNASEH2B*, *RNASEH2C*, and *SAMHD1*) have been linked to AGS and are related to an inability to remove cellular nucleotide debris intracellularly (Ali et al., 2006; Crow et al., 2006; Rice et al., 2009; Thiele et al., 2010; Xin et al., 2011). There is no direct link between LINE-1 activity and the cellular immune response; however, it has recently been suggested that LINE-1 activity is linked to both interferon expression and selected autoimmune disorders (Crow, 2010; Mavragani and Crow, 2010). A recent report concluded that LINE-1 activity is upregulated in *TREX1* knockout cells, suggesting that TREX1 is a LINE-1 inhibitor (Stetson et al., 2008). Here, we provide evidence that another AGS-related protein, SAMHD1, is also a potent LINE-1 suppressor. AGS-related mutations compromise the potency of SAMHD1 against LINE-1 retrotransposition. AGS-related mutations also compromised TREX1's potency against LINE-1 (Stetson et al., 2008). Interestingly, reverse transcriptase inhibitors could reduce disease symptoms generated in *TREX1*-knockout mice (Beck-Engeser et al., 2011). Further investigation is required to understand the relationship between retroelements such as LINE-1, AGS-suspected genes, and AGS itself.

EXPERIMENTAL PROCEDURES

Cell Culture and LINE-1 Retrotransposition Assay

HEK293T cells and HeLa-HA cells were grown in DMEM medium with 10% FBS (HyClone), GlutaMax, and Pen-strep (Invitrogen). All transfections used

Cell
PRESS
Open
ACCESS

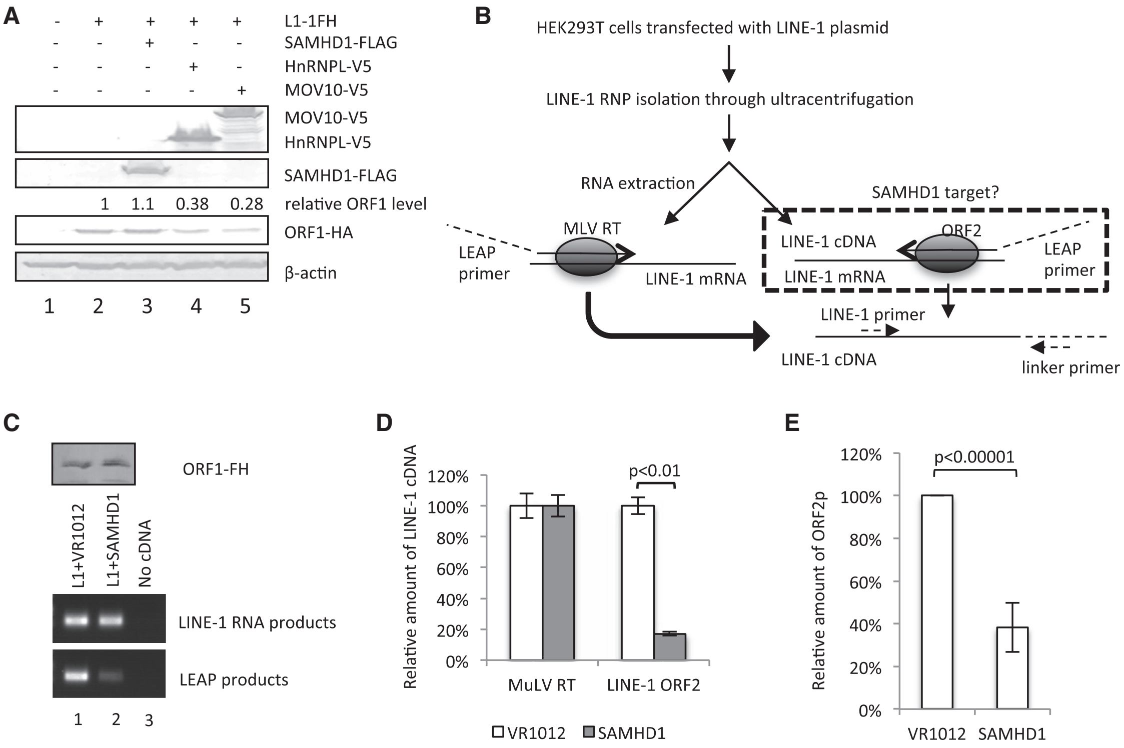

Figure 4. SAMHD1 Inhibits LINE-1 ORF2p-Mediated Endogenous Reverse Transcription in LINE-1 RNP

(A) SAMHD1 did not affect the expression of the LINE-1 ORF1 protein. HEK293T cells were transfected with the pc-L1-1FH vector plus the empty vector VR1012 or the expression vector for SAMHD1, HnRNPL, or MOV10. ImageJ software (NIH) was used to quantitate ORF1 band intensities, and their absolute readings are indicated above the immunoblot.

(B) A diagram of the LEAP assay. The LINE-1 RNP (from pc-L1-1FH) was produced from transfected HEK293T cells and purified by ultracentrifugation through a sucrose cushion as previously described. The LEAP primer, containing a linker region (dashed line), was used to precisely target onto LINE-1 mRNA, and the reverse transcription occurred with the assistance of the ORF2 protein. Synthesized cDNA was then amplified through standard PCR with two primers (dash arrows) targeting to LINE-1 and the linker.

(C) SAMHD1 reduced the reverse transcription efficiency mediated by ORF2p in LINE-1 RNPs. The amount of ORF1 proteins in isolated LINE-1 RNPs in the absence or presence of SAMHD1 was determined by immunoblotting using an anti-HA antibody. LINE-1 RNA was examined by using the LEAP primer and MuLV reverse transcriptase for cDNA synthesis, followed by PCR amplification.

(D) Quantitative real-time PCR analysis of LEAP products and LINE-1 RNA RT-PCR products.

(E) ORF2p level was lowered by 62% in average, with the presence of exogenous SAMDH1 protein. The bar chart was based on five independent experiments. The error bars indicated the SD.

See also Figure S4.

Lipofectamine 2000 (Invitrogen) reagent. HeLa-HA cells were a gift from J.V. Moran (University of Michigan).

The human L1 plasmids 99 PUR L1RP EGFP (L1) and 99 PUR JM111 EGFP (JM111, as a negative control) have been previously described (Ostertag et al., 2000). Similar protocols were also applied to either the synthetic human LINE-1 sL1-ORFeus-HS (An et al., 2011) or the murine LINE-1 plasmid ORFeus (gifts of Dr. J.D. Boeke) (Han and Boeke, 2004). In brief, LINE-1 plasmid was transfected into HEK293T cells at 2 μg per well in 12-well plates, together with VR1012 or one of the test plasmids. The cells were selected by the addition of puromycin (final concentration, 5 μg/ml) at 48 hr posttransfection. GFP-positive cells were examined 48 hr later by flow cytometry using FACSCalibur. Gating exclusions were based on background fluorescence of the plasmid 99 PUR JM111 EGFP, an L1 construct containing two point mutations in ORF1 that completely abolish retrotransposition; 10,000 single-cell events per sample were gated and analyzed using CellQuest Pro (v.5.2). For the mneol-based LINE-1 retrotransposition assay, 1 μg LcRPS-mneol was cotransfected in 6-well plates with 0.5 μg empty vector or the SAMHD1 expression vector into HEK293T cells. At 4 days posttransfection, selection with G418 was initiated and continued for 13 days. The cells in T75 flasks were fixed with PBS/paraformaldehyde/glutaraldehyde, and colonies stained with 0.4% Giemsa.

LEAP Assays and RT–PCR

The LINE-1 construct containing FLAG-HA-tagged ORF1, pc-L1-1FH, has been described (Goodier et al., 2012). It was cotransfected in the absence or presence of the SAMHD1 expression vector into HEK293T cells. At 2 days after transfection, LINE-1 RNPs were isolated by ultracentrifugation through a sucrose cushion as previously described (Kulpa and Moran, 2006). The LINE-1 RNP sample (2 μl) was added to each cDNA extension reaction (LEAP) as previously described (Kulpa and Moran, 2006), using the 3′ RACE adaptor NV: 5′-GCGAGCACAGAATTAATACGACTCACTATAGG TTTTTTTTTTTTVN-3′ as primer. Also, LINE-1 RNA was extracted from the LINE-1 RNP, treated with TURBO DNase (Invitrogen), and reverse transcribed using 3′ RACE adaptor NV as primer and MuLV RT with a High Capacity cDNA Reverse Transcription Kit (ABI Applied Biosystems). PCR was performed as previously described (Kulpa and Moran, 2006). PCR products were separated on 2% agarose gels and visualized by the Red Personal Gel Imaging System (ProteinSimple). PCR products were also sequenced and found to match the

expected LINE-1 sequence. The relative amount of synthesized cDNA from both methods were then determined by real-time PCR using the primers Linker (as part of primer 3′RACE adaptor NV), 5′-GCGAGCACAGAATTAATACGACT-3′; L1-LEAP-R, 5′-GGGTTCGAAATCGATAAGCTTGGATCCAGAC-3′, with a standard two step method (95°C for 15 s and 60°C for 1 min) with a cycle number of 40. See also Extended Experimental Procedures.

SUPPLEMENTAL INFORMATION

Supplemental Information includes Extended Experimental Procedures and four figures and can be found with this article online at http://dx.doi.org/10.1016/j.celrep.2013.08.019.

ACKNOWLEDGMENTS

We thank Drs. J. Boeke, D. Hancks, T. Inoue, M. Stevenson, and R. Siliciano for critical reagents; Y. Rui, W. Zheng, H. Guo, and J. Hou for technical assistance; R. Markham, J. Margolick, and J. Bream for thoughtful discussions; and D. McClellan for editorial assistance. We also wish to thank staff within the Mass Spectrometry Core and the Institute for Basic Biomedical Sciences Microscope Facility at Johns Hopkins School of Medicine for their technical assistance. The following reagent was obtained through the AIDS Research and Reference Reagents Program, Division of AIDS, NIAID, NIH: pHEF-VSVG (L.-J. Chang). This work was supported in part by funding from the Chinese Ministry of Science and Technology (2012CB911100 and No. 2013ZX0001-005) and Chinese Ministry of Education (IRT1016), the Key Laboratory of Molecular Virology, Jilin Province (20102209), China, a grant (2R56AI62644-6) from the NIAID, and a grant (1RC4MH092880-01) from the NIMH to H.H.K. K.Z., J.D., X.Z., J.L.G., P.L., W.W., S.L.E., X.H., L.L., and L.E.C. performed experiments. K.Z., J.D., J.L.G., L.E.C., W.Z., G.W., H.H.K., and X.-F.Y. analyzed the data. K.Z. and X.-F.Y. wrote the paper with help from all authors. X.-F.Y. directed the project.

Received: April 25, 2013
Revised: June 25, 2013
Accepted: August 15, 2013
Published: September 12, 2013

REFERENCES

Ali, M., Highet, L.J., Lacombe, D., Goizet, C., King, M.D., Tacke, U., van der Knaap, M.S., Lagae, L., Rittey, C., Brunner, H.G., et al. (2006). A second locus for Aicardi-Goutieres syndrome at chromosome 13q14-21. J. Med. Genet. *43*, 444–450.

An, W., Dai, L., Niewiadomska, A.M., Yetil, A., O'Donnell, K.A., Han, J.S., and Boeke, J.D. (2011). Characterization of a synthetic human LINE-1 retrotransposon ORFeus-Hs. Mob. DNA *2*, 2.

Aravind, L., and Koonin, E.V. (1998). The HD domain defines a new superfamily of metal-dependent phosphohydrolases. Trends Biochem. Sci. *23*, 469–472.

Baldauf, H.M., Pan, X., Erikson, E., Schmidt, S., Daddacha, W., Burggraf, M., Schenkova, K., Ambiel, I., Wabnitz, G., Gramberg, T., et al. (2012). SAMHD1 restricts HIV-1 infection in resting CD4(+) T cells. Nat. Med. *18*, 1682–1687.

Beck, C.R., Garcia-Perez, J.L., Badge, R.M., and Moran, J.V. (2011). LINE-1 elements in structural variation and disease. Annu. Rev. Genomics Hum. Genet. *12*, 187–215.

Beck-Engeser, G.B., Eilat, D., and Wabl, M. (2011). An autoimmune disease prevented by anti-retroviral drugs. Retrovirology *8*, 91.

Becker, K.G., Swergold, G.D., Ozato, K., and Thayer, R.E. (1993). Binding of the ubiquitous nuclear transcription factor YY1 to a cis regulatory sequence in the human LINE-1 transposable element. Hum. Mol. Genet. *2*, 1697–1702.

Berger, A., Sommer, A.F., Zwarg, J., Hamdorf, M., Welzel, K., Esly, N., Panitz, S., Reuter, A., Ramos, I., Jatiani, A., et al. (2011). SAMHD1-deficient CD14+ cells from individuals with Aicardi-Goutières syndrome are highly susceptible to HIV-1 infection. PLoS Pathog. *7*, e1002425.

Brouha, B., Schustak, J., Badge, R.M., Lutz-Prigge, S., Farley, A.H., Moran, J.V., and Kazazian, H.H., Jr. (2003). Hot L1s account for the bulk of retrotransposition in the human population. Proc. Natl. Acad. Sci. USA *100*, 5280–5285.

Cribier, A., Descours, B., Valadao, A.L., Laguette, N., and Benkirane, M. (2013). Phosphorylation of SAMHD1 by cyclin A2/CDK1 regulates its restriction activity toward HIV-1. Cell Reports *3*, 1036–1043.

Crow, M.K. (2010). Long interspersed nuclear elements (LINE-1): potential triggers of systemic autoimmune disease. Autoimmunity *43*, 7–16.

Crow, Y.J., Hayward, B.E., Parmar, R., Robins, P., Leitch, A., Ali, M., Black, D.N., van Bokhoven, H., Brunner, H.G., Hamel, B.C., et al. (2006). Mutations in the gene encoding the 3′-5′ DNA exonuclease TREX1 cause Aicardi-Goutières syndrome at the AGS1 locus. Nat. Genet. *38*, 917–920.

Dai, L., Taylor, M.S., O'Donnell, K.A., and Boeke, J.D. (2012). Poly(A) binding protein C1 is essential for efficient L1 retrotransposition and affects L1 RNP formation. Mol. Cell. Biol. *32*, 4323–4336.

Dale, R.C., Gornall, H., Singh-Grewal, D., Alcausin, M., Rice, G.I., and Crow, Y.J. (2010). Familial Aicardi-Goutières syndrome due to SAMHD1 mutations is associated with chronic arthropathy and contractures. Am. J. Med. Genet. A. *152A*, 938–942.

Descours, B., Cribier, A., Chable-Bessia, C., Ayinde, D., Rice, G., Crow, Y., Yatim, A., Schwartz, O., Laguette, N., and Benkirane, M. (2012). SAMHD1 restricts HIV-1 reverse transcription in quiescent CD4(+) T-cells. Retrovirology *9*, 87.

Feng, Q., Moran, J.V., Kazazian, H.H., Jr., and Boeke, J.D. (1996). Human L1 retrotransposon encodes a conserved endonuclease required for retrotransposition. Cell *87*, 905–916.

Goldstone, D.C., Ennis-Adeniran, V., Hedden, J.J., Groom, H.C., Rice, G.I., Christodoulou, E., Walker, P.A., Kelly, G., Haire, L.F., Yap, M.W., et al. (2011). HIV-1 restriction factor SAMHD1 is a deoxynucleoside triphosphate triphosphohydrolase. Nature *480*, 379–382.

Goodier, J.L., Cheung, L.E., and Kazazian, H.H., Jr. (2012). MOV10 RNA helicase is a potent inhibitor of retrotransposition in cells. PLoS Genet. *8*, e1002941.

Han, J.S., and Boeke, J.D. (2004). A highly active synthetic mammalian retrotransposon. Nature *429*, 314–318.

Hancks, D.C., and Kazazian, H.H., Jr. (2012). Active human retrotransposons: variation and disease. Curr. Opin. Genet. Dev. *22*, 191–203.

Harris, C.R., Dewan, A., Zupnick, A., Normart, R., Gabriel, A., Prives, C., Levine, A.J., and Hoh, J. (2009). p53 responsive elements in human retrotransposons. Oncogene *28*, 3857–3865.

Hrecka, K., Hao, C., Gierszewska, M., Swanson, S.K., Kesik-Brodacka, M., Srivastava, S., Florens, L., Washburn, M.P., and Skowronski, J. (2011). Vpx relieves inhibition of HIV-1 infection of macrophages mediated by the SAMHD1 protein. Nature *474*, 658–661.

Kim, B., Nguyen, L.A., Daddacha, W., and Hollenbaugh, J.A. (2012). Tight interplay among SAMHD1 protein level, cellular dNTP levels, and HIV-1 proviral DNA synthesis kinetics in human primary monocyte-derived macrophages. J. Biol. Chem. *287*, 21570–21574.

Kimberland, M.L., Divoky, V., Prchal, J., Schwahn, U., Berger, W., and Kazazian, H.H., Jr. (1999). Full-length human L1 insertions retain the capacity for high frequency retrotransposition in cultured cells. Hum. Mol. Genet. *8*, 1557–1560.

Kulpa, D.A., and Moran, J.V. (2006). Cis-preferential LINE-1 reverse transcriptase activity in ribonucleoprotein particles. Nat. Struct. Mol. Biol. *13*, 655–660.

Laguette, N., Sobhian, B., Casartelli, N., Ringeard, M., Chable-Bessia, C., Ségéral, E., Yatim, A., Emiliani, S., Schwartz, O., and Benkirane, M. (2011). SAMHD1 is the dendritic- and myeloid-cell-specific HIV-1 restriction factor counteracted by Vpx. Nature *474*, 654–657.

Lahouassa, H., Daddacha, W., Hofmann, H., Ayinde, D., Logue, E.C., Dragin, L., Bloch, N., Maudet, C., Bertrand, M., Gramberg, T., et al. (2012). SAMHD1 restricts the replication of human immunodeficiency virus type 1 by depleting the intracellular pool of deoxynucleoside triphosphates. Nat. Immunol. *13*, 223–228.

Lander, E.S., Linton, L.M., Birren, B., Nusbaum, C., Zody, M.C., Baldwin, J., Devon, K., Dewar, K., Doyle, M., FitzHugh, W., et al.; International Human Genome Sequencing Consortium. (2001). Initial sequencing and analysis of the human genome. Nature *409*, 860–921.

Luan, D.D., Korman, M.H., Jakubczak, J.L., and Eickbush, T.H. (1993). Reverse transcription of R2Bm RNA is primed by a nick at the chromosomal target site: a mechanism for non-LTR retrotransposition. Cell *72*, 595–605.

Manel, N., and Littman, D.R. (2011). Hiding in plain sight: how HIV evades innate immune responses. Cell *147*, 271–274.

Mathias, S.L., Scott, A.F., Kazazian, H.H., Jr., Boeke, J.D., and Gabriel, A. (1991). Reverse transcriptase encoded by a human transposable element. Science *254*, 1808–1810.

Mavragani, C.P., and Crow, M.K. (2010). Activation of the type I interferon pathway in primary Sjogren's syndrome. J. Autoimmun. *35*, 225–231.

Moran, J.V., Holmes, S.E., Naas, T.P., DeBerardinis, R.J., Boeke, J.D., and Kazazian, H.H., Jr. (1996). High frequency retrotransposition in cultured mammalian cells. Cell *87*, 917–927.

Niewiadomska, A.M., Tian, C., Tan, L., Wang, T., Sarkis, P.T., and Yu, X.F. (2007). Differential inhibition of long interspersed element 1 by APOBEC3 does not correlate with high-molecular-mass-complex formation or P-body association. J. Virol. *81*, 9577–9583.

Ostertag, E.M., Prak, E.T., DeBerardinis, R.J., Moran, J.V., and Kazazian, H.H., Jr. (2000). Determination of L1 retrotransposition kinetics in cultured cells. Nucleic Acids Res. *28*, 1418–1423.

Ostertag, E.M., Goodier, J.L., Zhang, Y., and Kazazian, H.H., Jr. (2003). SVA elements are nonautonomous retrotransposons that cause disease in humans. Am. J. Hum. Genet. *73*, 1444–1451.

Peddigari, S., Li, P.W., Rabe, J.L., and Martin, S.L. (2013). hnRNPL and nucleolin bind LINE-1 RNA and function as host factors to modulate retrotransposition. Nucleic Acids Res. *41*, 575–585.

Powell, R.D., Holland, P.J., Hollis, T., and Perrino, F.W. (2011). Aicardi-Goutieres syndrome gene and HIV-1 restriction factor SAMHD1 is a dGTP-regulated deoxynucleotide triphosphohydrolase. J. Biol. Chem. *286*, 43596–43600.

Rice, G.I., Bond, J., Asipu, A., Brunette, R.L., Manfield, I.W., Carr, I.M., Fuller, J.C., Jackson, R.M., Lamb, T., Briggs, T.A., et al. (2009). Mutations involved in Aicardi-Goutières syndrome implicate SAMHD1 as regulator of the innate immune response. Nat. Genet. *41*, 829–832.

Stetson, D.B., Ko, J.S., Heidmann, T., and Medzhitov, R. (2008). Trex1 prevents cell-intrinsic initiation of autoimmunity. Cell *134*, 587–598.

Tchénio, T., Casella, J.F., and Heidmann, T. (2000). Members of the SRY family regulate the human LINE retrotransposons. Nucleic Acids Res. *28*, 411–415.

Thiele, H., du Moulin, M., Barczyk, K., George, C., Schwindt, W., Nürnberg, G., Frosch, M., Kurlemann, G., Roth, J., Nürnberg, P., and Rutsch, F. (2010). Cerebral arterial stenoses and stroke: novel features of Aicardi-Goutières syndrome caused by the Arg164X mutation in SAMHD1 are associated with altered cytokine expression. Hum. Mutat. *31*, E1836–E1850.

Wang, H., Xing, J., Grover, D., Hedges, D.J., Han, K., Walker, J.A., and Batzer, M.A. (2005). SVA elements: a hominid-specific retroposon family. J. Mol. Biol. *354*, 994–1007.

Wei, W., Guo, H., Han, X., Liu, X., Zhou, X., Zhang, W., and Yu, X.F. (2012). A novel DCAF1-binding motif required for Vpx-mediated degradation of nuclear SAMHD1 and Vpr-induced G2 arrest. Cell. Microbiol. *14*, 1745–1756.

White, T.E., Brandariz-Nuñez, A., Valle-Casuso, J.C., Amie, S., Nguyen, L., Kim, B., Brojatsch, J., and Diaz-Griffero, F. (2013a). Contribution of SAM and HD domains to retroviral restriction mediated by human SAMHD1. Virology *436*, 81–90.

White, T.E., Brandariz-Nuñez, A., Valle-Casuso, J.C., Amie, S., Nguyen, L.A., Kim, B., Tuzova, M., and Diaz-Griffero, F. (2013b). The retroviral restriction ability of SAMHD1, but not its deoxynucleotide triphosphohydrolase activity, is regulated by phosphorylation. Cell Host Microbe *13*, 441–451.

Xin, B., Jones, S., Puffenberger, E.G., Hinze, C., Bright, A., Tan, H., Zhou, A., Wu, G., Vargus-Adams, J., Agamanolis, D., and Wang, H. (2011). Homozygous mutation in SAMHD1 gene causes cerebral vasculopathy and early onset stroke. Proc. Natl. Acad. Sci. USA *108*, 5372–5377.

Yang, N., Zhang, L., Zhang, Y., and Kazazian, H.H., Jr. (2003). An important role for RUNX3 in human L1 transcription and retrotransposition. Nucleic Acids Res. *31*, 4929–4940.

Cell PRESS Open ACCESS

EXTENDED EXPERIMENTAL PROCEDURES

Plasmid Construction

The SAMHD1 gene was retrieved from human blood through reverse transcription followed by polymerase chain reaction, and then subcloned into VR1012 (Hartikka et al., 1996; Zhang et al., 2012) (containing a CMV promoter/enhancer, intronA, multiple cloning sites, BGH polyA signal sequence), or pmCherry-C1 (Clonetech, a gift of Dr. T. Inoue), with an HA or FLAG tag at the C terminus. Various site mutations in SAMHD1 and truncation mutants of SAMHD1were introduced using standard site-directed mutagenesis techniques. All mutant constructs were sequence-confirmed. Non-human SAMHD1 expression vectors were obtained from Boyi Biotech Co., Ltd (Changchun, China). TREX1 was also obtained from human blood and subcloned into VR1012 with a FLAG tag at the C terminus. 99 PUR RPS EGFP (Ostertag et al., 2000), 99 PUR JM111 EGFP (Ostertag et al., 2000), pc-RP-1FH (Goodier et al., 2012), Alu neo^{Tet} (Dewannieux et al., 2003), and SVA.2 EGFP (Hancks et al., 2011) retrotransposition reporter constructs have been described.

Antibodies

The following antibodies were used to detect protein expression: anti-SAMHD1 and anti-tubulin from Abcam (Cambridge, MA), anti-HA and anti-V5 from Invitrogen (Carlsbad, CA), anti-β-actin from GenScript (Piscataway, NJ), anti-FLAG from Sigma, and anti-c-*myc* from Millipore (Billerica, MA). Anti-ORF2p antibody was used as previously described (Goodier et al., 2012). All antibodies were used according to the manufacturers' protocols.

Modification of SAMHD1 Expression by siRNA Silencing and Vpx Expression

RNAi against SAMHD1 was carried out using a pool of four duplexed short interfering RNAs (siRNAs) (Dharmacon, smart pool: L-013950-01): J-013950-09: 5′-GACAAUGAGUUGCGUAUUU-3′; J-013950-10: 5′-CAUGUUUGAUGGACGAAUUU-3′; J-013950-11: 5′-AAGUAUUGCUAGACGUGAA-3′ and J-013950-12: 5′-UUAGUUAUAUCCAGCGAUU-3′. Human embryonic kidney HEK293T cells were transfected with the SAMHD1 siRNA pool at a total final concentration of 100 nM using Lipofectamine 2000 (Invitrogen). The nontargeting siRNA no. 2 (Dharmacon) was used as a control. SAMHD1 protein expression was monitored by immunoblotting 2 days after transfection using SAMHD1 specific antibody from Abcam. SAMHD1 mRNA was analyzed by quantitative real-time PCR (qRT-PCR). RNA obtained from cells was purified by Trizol (Life Technology) according to the manufacturer's protocol and treated with DNase I by incubation in 10 μl of diethyl pyrocarbonate (DEPC)-treated water with 1x RQ1 RNase-Free DNase buffer, l μl of RQ1 RNase-free DNase (Promega), and 4 U of RNase inhibitor (New England Biolabs) for 30 min at 37°C. The DNase was inactivated by the addition of 1 μl RQ1 DNase stop solution and incubation at 65°C for 10 min. The RNA was reverse-transcribed by using random primers and the Multiscribe reverse transcriptase from the High-Capacity cDNA Archive Kit (Applied Biosystems) according to the manufacturer's instructions. The cDNA was either undiluted or serially diluted in DEPC-treated water before input into the real-time reaction to ensure that the amplification was within the linear range of detection. The StepOne Real-Time PCR system (Applied Biosystems, Carlsbad, CA) was used for the qRT-PCR amplifications. The reactions were performed under the following conditions: 50°C for 2 min and 95°C for 10 min, followed by 40 cycles of 95°C for 15 s and 60°C for 1 min, followed by a dissociation protocol. Single peaks in the melting curve analysis indicated specific amplicons. The target sequences were amplified using the following primer pairs: GAPDH, forward (5′-GCAAATTCCATGGCACCGT-3′) and reverse (5′-TCGCCCCACTTGATTTTGG-3′); SAMHD1, forward (5′-TCCATCCCGACTACAAGACA-3′) and reverse (5′-TCTCGGATGTTCTTCAGCAG-3′). To reduce SAMHD1 protein expression, Vpx and VpxQ76A expression vectors were transfected into HEK293T cells as previously described (Wei et al., 2012), and SAMHD1 expression was analyzed by immunoblotting using SAMHD1-specific antibody from Abcam.

Retroviral Production and Infection

SIV_{WT}GFP, $SIV_{\Delta Vpx}$GFP (both gifts of Dr. M. Stevenson) (Sharova et al., 2008), or NL43-ΔE-EGFP (a generous gift of Dr. R. Siliciano) (Zhang et al., 2012) was cotransfected together with pHEF-VSVG (from Dr. L.-J. Chang, through the AIDS Research and Reference Reagent Program, Division of AIDS, NIAID, NIH) (Chang et al., 1999) into HEK293T cells by using Lipofectamine 2000 (Invitrogen) at a concentration suggested by the manufacturer's protocol. The medium was changed at 24 hr post-transfection, and the supernatant was collected after an additional 24 hr. After the cells and debris were removed by filtration, the virus titers were standardized by Magi infection (Chackerian et al., 1997). For viral infection, equal amounts of viruses were used to infect HEK293T cells seeded onto a 12-well plate in the presence of DEAE (Sigma) at a final concentration of 20 μg/ml. The cells were collected and analyzed for GFP expression using a FACSCalibur (BD Biosciences, San Jose, CA); 10,000 single-cell events per sample were gated and analyzed using CellQuest Pro (v.5.2).

HBV Inhibition

The expression vector of wild-type human SAMHD1 was cotransfected with 2 μg HBV-expressing vector (Bouchard et al., 2001) into HEK293T cells. The empty vector VR1012 was used as a negative control for the effect of SAMHD1. The HBsAg concentration in the culture medium was determined at 48 hr post-transfection by ELISA using an EIA kit for the detection of HBsAg (Kehua Bio-engineering Co., LTD, Shanghai, China). Culture medium from transfected cells containing no HBV expression vector was used as negative control for HBsAg detection.

SVA and Alu Retrotransposition Assay

SVA retrotransposition assays were conducted as previously described (Hancks et al., 2011). pCEP Pur SVA.2 EGFP (SVA.2-EGFP) and pcDNA6.ORF2 (1 μg each) were cotransfected with the empty vector VR1012 or the VR1012-SAMHD1 expression vector into HEK293T cells seeded on a 12-well plate. Transfected cells were then selected at 48 hr post-transfection by adding puromycin at 5 μg/ml. Selected cells were tested for GFP expression by flow cytometry using FACSCalibur after another 48 hr. Gating exclusions were based on the background fluorescence of plasmid SVA.2-EGFP plus pcDNA3; 100,000 single-cell events per sample were gated and analyzed using CellQuest Pro (v.5.2).

The Alu retrotransposition assays were carried out essentially as described in Dewannieux et al.(Dewannieux et al., 2003). The retrotransposition construct Alu-*neo*^{Tet}, containing a Ya5 subfamily Alu and neomycin phosphotransferase reporter gene interrupted by an inverted self-splicing Group I intron, was cotransfected with pcDNA3 empty vector or a retrotransposition driver plasmid expressing L1 ORF2p (pCEP 5′ UTR ORF2 No Neo (Alisch et al., 2006), a gift of J.L. García-Pérez, Pfizer University of Granada). HeLa-HA cells were expanded from six-well plates to T75 flasks, and at 5 days post-transfection 600 μg/ml of G418 were added to select for retrotransposition events. After 15 days of selection, the cells were fixed and stained with Giemsa, and colonies were counted.

Statistical analysis (Student's t test, two-tailed) was performed with Microsoft Excel.

LEAP Assays and RT-PCR

The LINE-1 construct containing FLAG-HA-tagged ORF1, pc-L1-1FH, has been described (Goodier et al., 2012). It was cotransfected in the absence or presence of the SAMHD1 expression vector into HEK293T cells. At 2 days after transfection, LINE-1 RNPs were isolated by ultracentrifugation through a sucrose cushion as previously described (Kulpa and Moran, 2006). The LINE-1 RNP sample (2 μl) was added to each cDNA extension reaction (LEAP) as previously described (Kulpa and Moran, 2006), using the 3′RACE adaptor NV: 5′-GCGAGCACAGAATTAATACGACTCACTATAGGTTTTTTTTTTTTVN-3′ as primer. Also, LINE-1 RNA was extracted from the LINE-1 RNP, treated with TURBO DNase (Invitrogen), and reverse-transcribed using 3′RACE adaptor NV as primer and MuLV RT with a High Capacity cDNA Reverse Transcription Kit (ABI Applied Biosystems). PCR was performed as previously described (Kulpa and Moran, 2006). PCR products were separated on 2% agarose gels and visualized by the Red Personal Gel Imaging System (ProteinSimple). PCR products were also sequenced and found to match the expected LINE-1 sequence. The relative amount of synthesized cDNA from both methods were then determined by real-time PCR using the primers Linker (as part of primer 3′RACE adaptor NV), 5′-GCGAGCACAGAATTAATACGACT-3′; L1-LEAP-R, 5′-GGGTTCGAAATCGATAAGCTTGGATCC AGAC-3′, with a standard 2-step method (95°C for 15 s and 60°C for 1 min) with a cycle number of 40.

Live-Cell Imaging

Plasmid (pEYFP-Nuc [a gift of Dr. T. Inoue], 0.25 μg) and pmCherry-SAMHD1-HA (2 μg) were transfected into HEK293T cells using PEI Max (Polysciences) according to the manufacturer's protocol. For live-cell imaging, HEK293T cells were transfected in 6-well coverslip glass-bottomed cell culture dishes (InVitro Scientific) when the cells were ~80% confluent, and then visualized after 24 hr using a Zeiss LSM510-Meta confocal imaging system equipped with four argon lasers (458, 477, 488, and 514 nm lines), two HeNe lasers (542 and 633 nm), and one diode laser (405 nm). All images were acquired from a 63× objective, and image analysis and manipulation were performed using Zen 2009 software.

SUPPLEMENTAL REFERENCES

Alisch, R.S., Garcia-Perez, J.L., Muotri, A.R., Gage, F.H., and Moran, J.V. (2006). Unconventional translation of mammalian LINE-1 retrotransposons. Genes Dev. *20*, 210–224.

Bouchard, M.J., Wang, L.H., and Schneider, R.J. (2001). Calcium signaling by HBx protein in hepatitis B virus DNA replication. Science *294*, 2376–2378.

Chackerian, B., Long, E.M., Luciw, P.A., and Overbaugh, J. (1997). Human immunodeficiency virus type 1 coreceptors participate in postentry stages in the virus replication cycle and function in simian immunodeficiency virus infection. J. Virol. *71*, 3932–3939.

Chang, L.J., Urlacher, V., Iwakuma, T., Cui, Y., and Zucali, J. (1999). Efficacy and safety analyses of a recombinant human immunodeficiency virus type 1 derived vector system. Gene Ther. *6*, 715–728.

Dewannieux, M., Esnault, C., and Heidmann, T. (2003). LINE-mediated retrotransposition of marked Alu sequences. Nat. Genet. *35*, 41–48.

Goncalves, A., Karayel, E., Rice, G.I., Bennett, K.L., Crow, Y.J., Superti-Furga, G., and Bürckstümmer, T. (2012). SAMHD1 is a nucleic-acid binding protein that is mislocalized due to aicardi-goutières syndrome-associated mutations. Hum. Mutat. *33*, 1116–1122.

Hancks, D.C., Goodier, J.L., Mandal, P.K., Cheung, L.E., and Kazazian, H.H., Jr. (2011). Retrotransposition of marked SVA elements by human L1s in cultured cells. Hum. Mol. Genet. *20*, 3386–3400.

Hartikka, J., Sawdey, M., Cornefert-Jensen, F., Margalith, M., Barnhart, K., Nolasco, M., Vahlsing, H.L., Meek, J., Marquet, M., Hobart, P., et al. (1996). An improved plasmid DNA expression vector for direct injection into skeletal muscle. Hum. Gene Ther. *7*, 1205–1217.

Hofmann, H., Logue, E.C., Bloch, N., Daddacha, W., Polsky, S.B., Schultz, M.L., Kim, B., and Landau, N.R. (2012). The Vpx lentiviral accessory protein targets SAMHD1 for degradation in the nucleus. J. Virol. *86*, 12552–12560.

Sharova, N., Wu, Y., Zhu, X., Stranska, R., Kaushik, R., Sharkey, M., and Stevenson, M. (2008). Primate lentiviral Vpx commandeers DDB1 to counteract a macrophage restriction. PLoS Pathog. *4*, e1000057.

Zhang, W., Du, J., Evans, S.L., Yu, Y., and Yu, X.F. (2012). T-cell differentiation factor CBF-β regulates HIV-1 Vif-mediated evasion of host restriction. Nature *481*, 376–379.

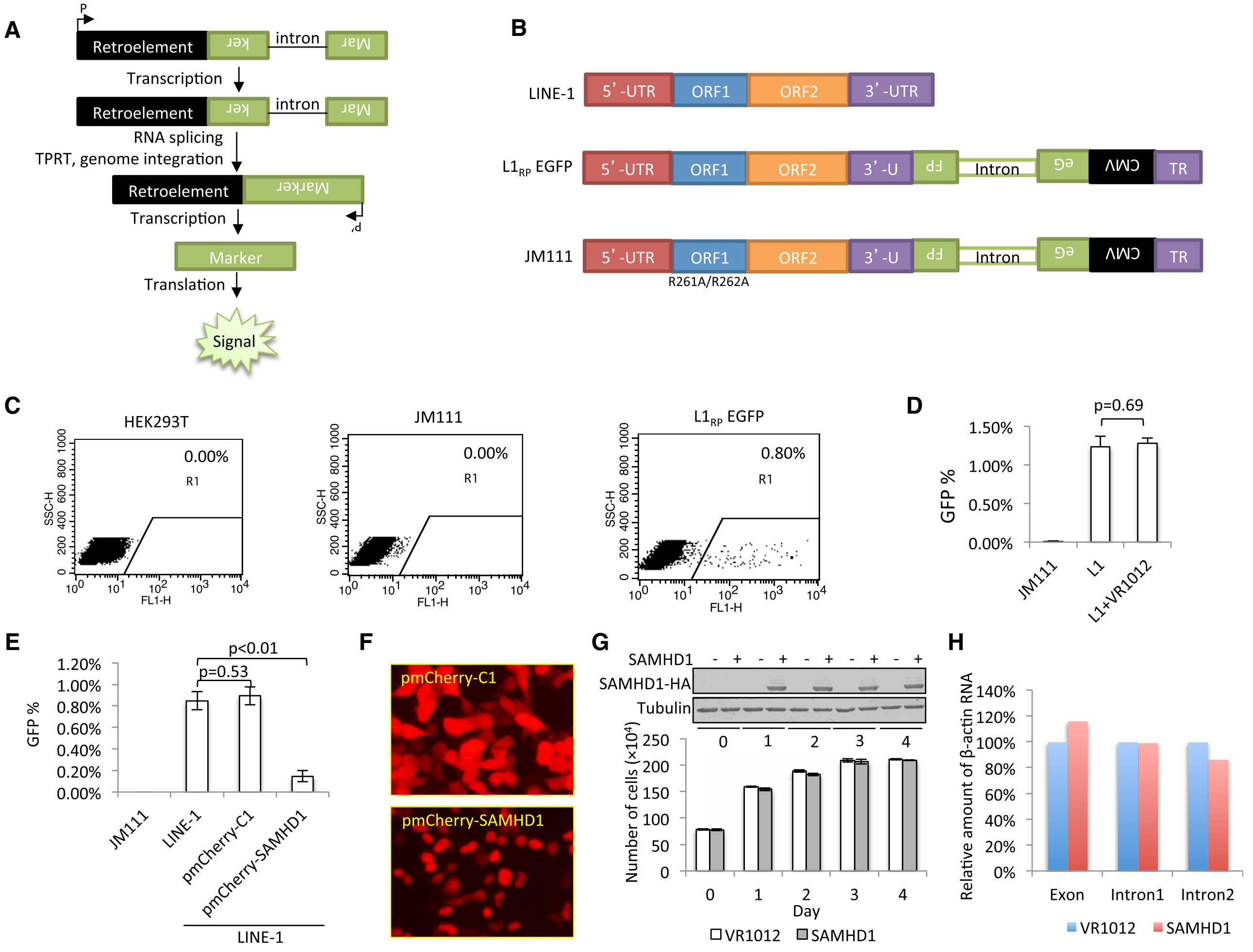

Figure S1. Modulation of LINE-1 (L_{RP} EGFP) Retrotransposition by SAMHD1 Is Not Related to Its Cytotoxicity, Related to Figure 1

(A) Process of retrotransposition assay. A marker can only be detected when retroelement successfully transposes onto genome with the intron removed during RNA splicing;

(B) Schematic of 99 PUR RPS EGFP (LINE-1) and JM111 EGFP (JM111), which have been previously described (Moran et al., 1996). The 99 PUR RPS EGFP contains combined promoters from both CMV and the 5’-UTR of the LINE-1. An antisense cassette of EGFP containing an inserted intron sequence was cloned near the 3’ end of the L1 3’ UTR. JM111 contains a double mutation of R261A/R262A in ORF1, which has been determined to abolish the retrotransposition activity of LINE-1.

(C) Representative flow cytometry data for both JM111 and LINE-1 construct in HEK293T cells. Comparing to retrotransposition-competent LINE-1, JM111 showed no EGFP signal and thus was used as negative control for the following experiment. 10,000 single cells was gated and percentage of EGFP-positive events was shown on up-right corner of each panel.

(D) The absence or presence of the empty vector VR1012 has no apparent effect on 99 PUR RPS EGFP activity in transfected HEK293T cells. The bar charts represent three independent experiments; error bars indicate the S.D. of three replicates within one experiment;

(E) CMV-promoted expression of irrelative protein mCherry has no effect against LINE-1 retrotransposition, while mCherry-fused SAMHD1 still presents strong potency against LINE-1 activity. The bar charts represent three independent experiments; error bars indicate the S.D. of three replicates within one experiment;

(F) Expression of mCherry and mCherry-fused SAMHD1 in HEK293T cells. Live-cell imaging indicated that wild type SAMHD1 were localized to the nucleus of HEK293T cells. mCherry was fused to the N-terminus of the SAMHD1 protein;

(G) SAMHD1 did not cause cytotoxicity to HEK293T cells. HEK293T cells were transfected with either a SAMHD1-expressing plasmid or the empty vector VR1012 as a negative control. Viable transfected cells were counted daily from Day 0 to Day 4. The bar charts are representative of three independent experiments; error bars indicate the S.D. of three replicates within one experiment.

(H) SAMHD1 does not affect intron splicing of β-actin pre-mRNA. The following primers were used to determine the relative amount of pre-mRNA of β–actin: Exon-F, 5’-TCACCCACACTGTGCCCATCTACGA-3’; Exon-R, 5’-CAGCGGAACCGCTCATTGCCAATGG-3’; Intron1-F, 5’-CCGTGCTCAGGGCTTCTT-3’; Intron1-R, 5’-CCATGTCGTCCCAGTTGGT-3’; Intron2-F, 5’-GGAGCTGTCACATCCAGGGTC-3’; Intron2-R, 5’-GCATTTGCGGTGGACGAT-3’.

Cell PRESS
Open ACCESS

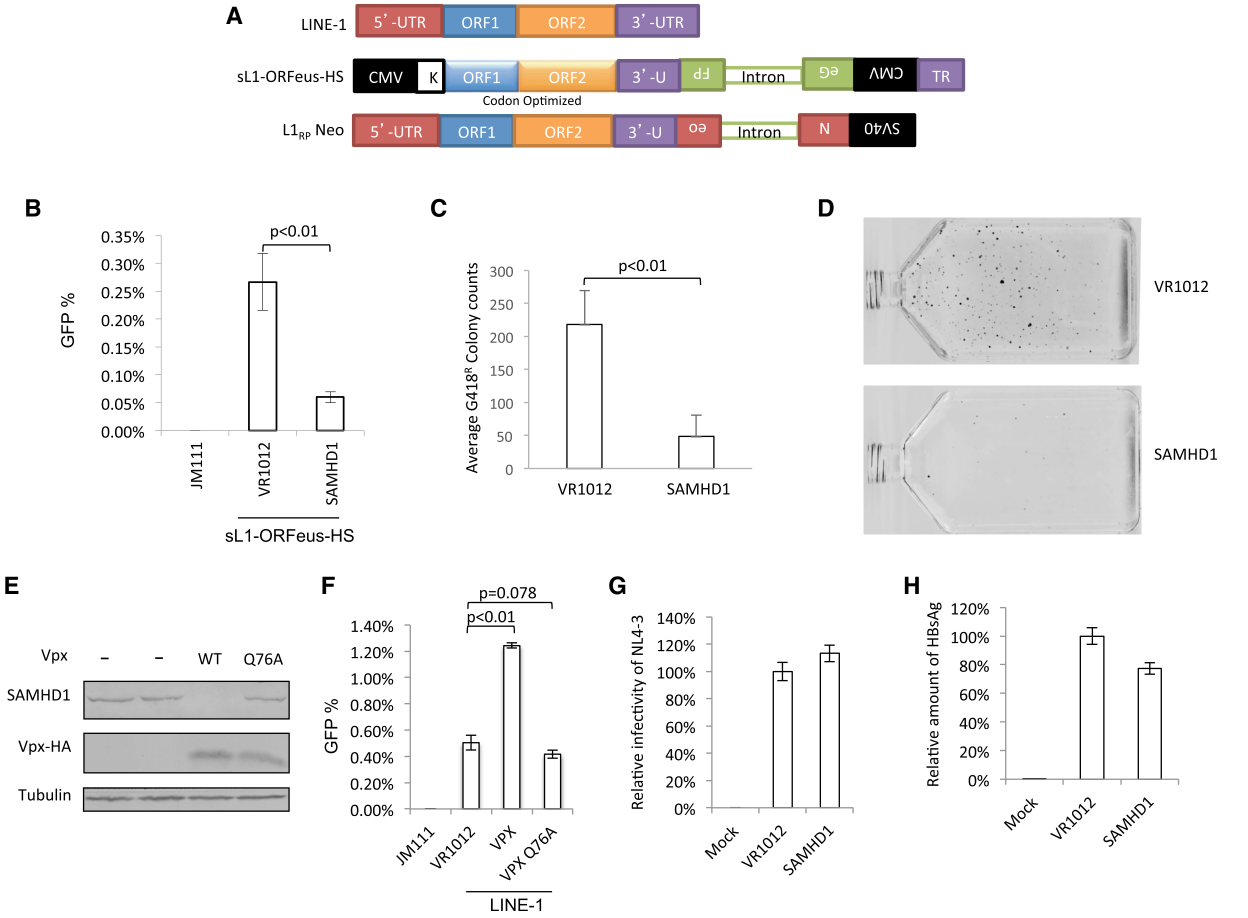

Figure S2. Modulation of Synthetic Human LINE-1 (sL1-ORFeus-HS) and mneol-Based Human LINE-1 ($L1_{RP}$ Neo) Retrotransposition by SAMHD1, Related to Figure 1

(A) Comparison between natural LINE-1, codon optimized sL1-ORFeus-HS and mneol-based LINE-1 constructs. The sL1-ORFeus-HS does not contain the 5'UTR of human LINE-1. The transcription of LINE-1 was driven by CMV promoter and LINE-1 protein translation was mediated from a Kozac sequence (acc). Both ORF1 and ORF2 coding regions were codon-optimized with 25% of the nucleotides altered. The 3'-UTR was separated by insertion of an antisense cassette of EGFP containing a sense intron. L1RP Neo is similar with L1RP EGFP, but with an antisense neomycin-resistance marker driven by SV40 promoter;

(B) SAMHD1 potently suppresses the activity of sL1-ORFeus-HS in HEK293T cells. The bar charts are representative of three independent experiments; error bars indicate the S.D. of three replicates within one experiment;

(C) SAMHD1 potently suppresses LINE-1 activity (using the neo gene as reporter). LINE-1 neo was co-transfected with VR1012 or SAMHD1 into HeLa-HA cells, which were subjected to G418 selection (600 μg/ml) for 2 weeks, then visualized through 0.4% Giemsa staining. The error bars indicate the S.D. of four replicates within one experiment;

(D) Representative flask for Supplementary Figure 2C;

(E) Expression of Vpx, but not the Vpx Q76A mutant, can deplete endogenous SAMHD1 in transfected HEK293T cells. The efficient depletion of endogenous SAMHD1 in transfected HEK293T cells is consistent with the results of 90% transfection efficiency in parallel experiments using EGFP expression as the indicator protein.

(F) Vpx, but not the Vpx Q76A mutant, can promote LINE-1 activity in HEK293T cells. HEK293T cells were transfected with L1RP-EGFP, together with Vpx or Vpx Q76A. Transfected cells were subjected to flow cytometry to determine the percentage of GFP-positive cells. VR1012 was used as a negative control for Vpx, and JM111 as a negative control for L1RP-EGFP. The bar charts are representative of three independent experiments; error bars indicate the S.D. of three replicates within one experiment;

(G) SAMHD1 did not inhibit the infection of NL4-3. VSVg-coated NL4-3 EGFP was used to infect HEK293T cells at 24 h post-transfection of VR1012 or SAMHD1. Infected cells were subjected to flow cytometry to determine the percentage of EGFP-positive cells. Relative infectivity is shown. The bar charts represent three independent experiments; error bars indicate the S.D. of three replicates within one experiment;

(H) SAMHD1 barely affects the production of HBV. The HBV-expressing vector was co-transfected with VR1012 or SAMHD1 into HEK293T cells. The amount of HBsAg in the supernatant of the culture was determined at 2 days post-transfected. Relative amounts are shown. The bar charts represent three independent experiments; error bars indicate the S.D. of three replicates within one experiment.

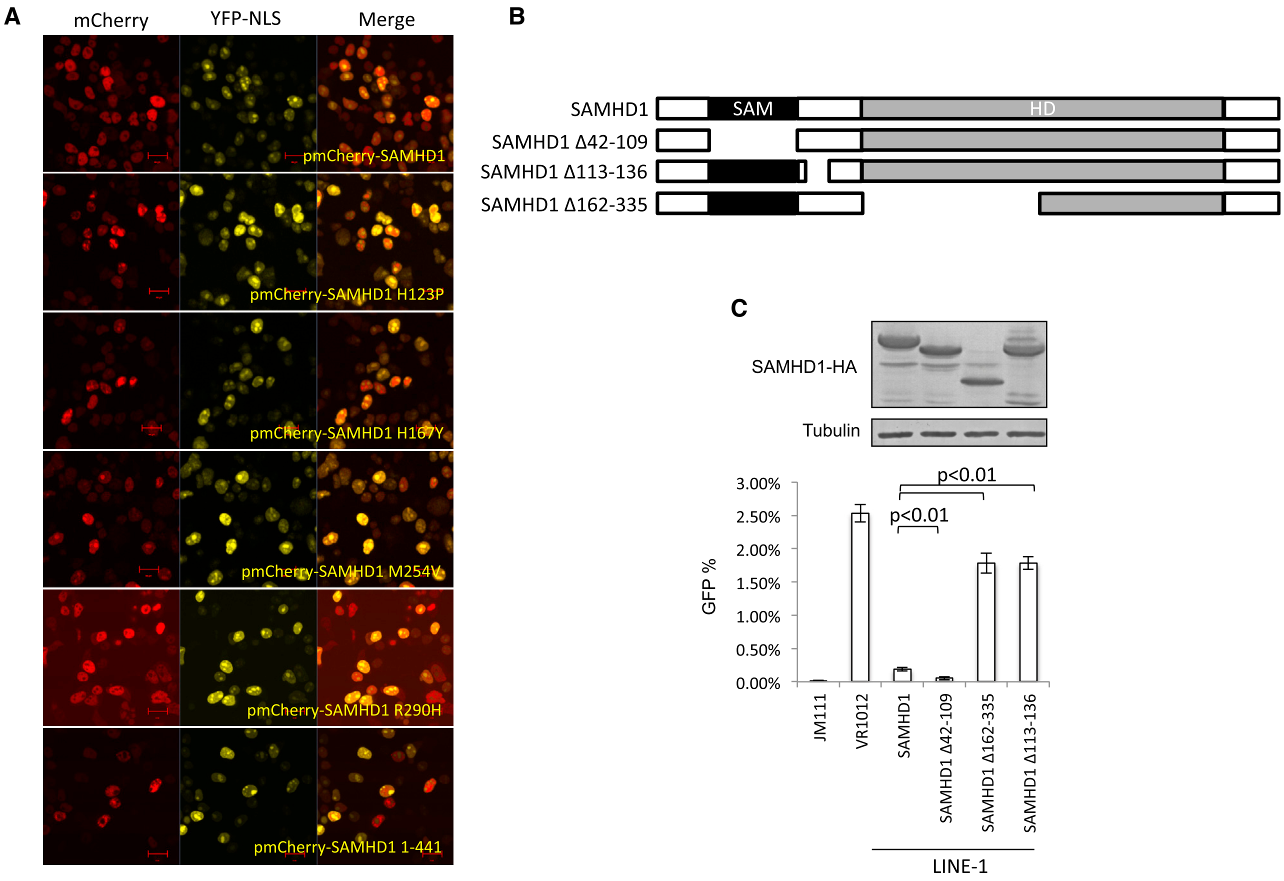

Figure S3. The Linker Region and HD Domain Are Important for SAMHD1-Mediated LINE-1 Inhibition, Related to Figure 2

(A) Cellular localization of mCherry-tagged SAMHD1 and SAMHD1 mutant proteins using live cell imaging. All tested AGS-related SAMHD1 mutants were localized in nuclei of HEK293T cells, which is different from a previous observation in HeLa cells (Goncalves et al., 2012). Different experimental conditions may have contributed to the different observations. A recent report also indicated that AGS-related SAMHD1 mutants were localized in nucleus (Hofmann et al., 2012). Plasmid pEYFP-Nuc (Clontech) and pmCherry-SAMHD1 (WT) or indicated mutants were co-transfected into HEK293T cells using PEI Max (Polysciences);

(B) Schematic of SAMHD1 domain truncations;

(C) Truncations in both the HD domain and linker region show poor potency against LINE-1 (bars 5 and 6), whereas the construct in which SAM domain is deleted retains strong LINE-1 inhibition activity (bar 4). The bar charts represent three independent experiments; error bars indicate the S.D. of three replicates within one experiment.

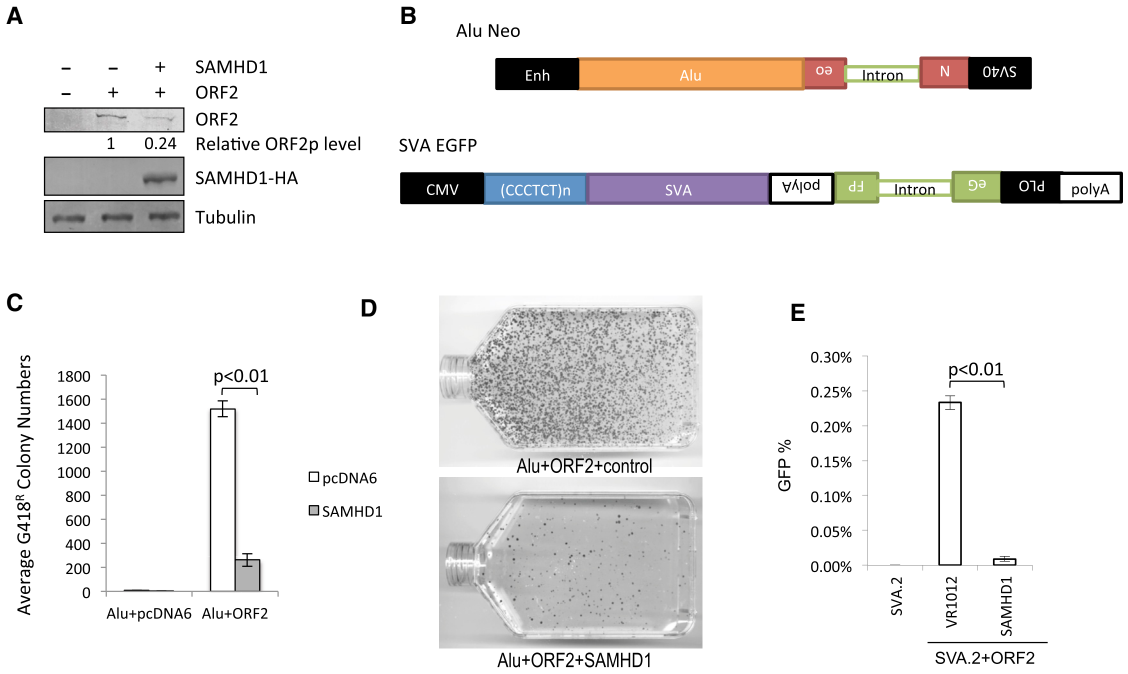

Figure S4. Modulation of Alu and SVA Retrotransposition by SAMHD1, Related to Figure 4

(A) Representative blot indicating expression/stability of ORF2p was compromised by the presence of SAMHD1 in HEK293T cells;

(B) Cartoon of mneoI-based Alu and EGFP-based SVA construct. The Ya5 Alu sequence is cloned into a plasmid containing the 7SL pol III enhancer and neoTET cassette interrupted by a Tetrahymena self-splicing intron;

(C) SAMHD1 potently suppresses ORF2p-mediated retrotransposition of Alu. HeLa cells were transfected with Alu-neoTET plus the empty vector pcDNA6 or the ORF2p expression vector. Transfected cells were treated with neomycin (600 μg/ml) for 15 days. Resistant colonies were stained with 0.4% Giemsa and counted. The error bars indicate the S.D. of four replicates within one experiment;

(D) Representative flask for Supplementary Figure 4C;

(E) SAMHD1 suppresses ORF2p-mediated retrotransposition of SVA. pCEP Pur SVA.2 EGFP (SVA.2-EGFP) and pcDNA6.ORF2 (1 μg each) were co-transfected with empty vector VR1012 or the VR1012-SAMHD1 expression vector into HEK293T cells seeded on a 12-well plate. Transfected cells were then selected at 48 h post-transfection by adding puromycin at 5 μg/mL. Selected cells were tested for GFP expression by flow cytometry using FACSCalibur after another 48 h. Gating exclusions were based on the background fluorescence of the plasmid SVA.2-EGFP plus pcDNA3; 100,000 single-cell events per sample were gated and analyzed using CellQuest Pro.
